# Urine lipoarabinomannan in HIV uninfected, smear negative, symptomatic TB patients: effective sample pretreatment for a sensitive immunoassay and mass spectrometry

**DOI:** 10.1038/s41598-021-82445-4

**Published:** 2021-02-03

**Authors:** Anita G. Amin, Prithwiraj De, Barbara Graham, Roger I. Calderon, Molly F. Franke, Delphi Chatterjee

**Affiliations:** 1 Mycobacteria Research Laboratory, Department of Microbiology, Immunology and Pathology, Colorado State University, Fort Collins, CO 80523 USA; 2 Socios en Salud Sucursal Peru, Lima, 15001 Peru; 3 Programa Academico de Tuberculose, Faculdade de Medicina, Universidade Federal do Rio de Janeiro, Rio de Janeiro, 21941-590 Brazil; 4 Department of Global Health and Social Medicine, Harvard Medical School, Boston, MA 02115 USA

**Keywords:** Microbiology, Biomarkers, Medical research, Immunological techniques, Mass spectrometry, Diseases, Infectious diseases, Tuberculosis

## Abstract

Our study sought to determine whether urine lipoarabinomannan (LAM) could be validated in a sample cohort that consisted mainly of HIV uninfected individuals that presented with tuberculosis symptoms. We evaluated two tests developed in our laboratory, and used them on clinical samples from Lima, Peru where incidence of HIV is low. ELISA analysis was performed on 160 samples (from 140 adult culture-confirmed TB cases and 20 symptomatic TB-negative child controls) using 100 μL of urine after pretreatment with Proteinase K. Two different mouse monoclonal antibodies-CS35 and CHCS9-08 were used individually for capture of urine LAM. Among cases, optical density (OD_450_) values had a positive association with higher bacillary loads. The 20 controls had negative values (below the limit of detection). The assay correctly identified all samples (97–100% accuracy confidence interval). For an alternate validation of the ELISA results, we analyzed all 160 urine samples using an antibody independent chemoanalytical approach. Samples were called positive only when LAM surrogates—tuberculostearic acid (TBSA) and d-arabinose (d-ara)—were found to be present in similar amounts. All TB cases, including the 40 with a negative sputum smear had LAM in detectable quantities in urine. None of the controls had detectable amounts of LAM. Our study shows that urinary LAM detection is feasible in HIV uninfected, smear negative TB patients.

## Introduction

Tuberculosis (TB) remains one of the leading causes of death worldwide. The number of new cases of TB has been declining steadily in recent years. However, the burden remains high among low-income and marginalized populations with about 10 million people developing TB in 2018^1^. The report also notes that an estimated 3 million of those with TB still are not getting the care they need. This could have been preventable if a sensitive and early diagnosis were available. Mycobacterial culture, the current gold diagnostic standard and conventional techniques for diagnosing TB normally take two to three weeks. In 2018, only 55% of pulmonary cases were bacteriologically confirmed. It is estimated that 42% of pulmonary TB (PTB) cases are smear negative^2^. The Xpert/RIF assay provides rapid detection of *Mycobacterium tuberculosis* nucleic acids from sputum and delivers a diagnostic accuracy of 79%, provided patients with HIV are able to expectorate^3,4^. This delay in diagnosis and further increase in detection complexity due to the emerging risks of XDR-TB (Extensively drug Resistant-TB) and MDR-TB (Multidrug Resistant-TB) are evoking interest of researchers in the field of developing rapid TB detection techniques. Early diagnosis and on-time effective treatment are indispensable for the control of TB—a life threatening infectious communicable disease.

In recent years, urinary lipoarabinomannan (LAM) based diagnostics for TB have gained significant advances^5–9^. Specimens like serum and urine collection are less invasive and can be easily collected from patients visiting or admitted to the hospital or even outside of the hospital setting (e.g. at home). Current commercial urinary LAM based diagnostic kits have a specificity of 95% or more and sensitivity of 40–70% but are recommended only for patients with HIV and CD4 counts less than 100 cells per microliter. One such test, Alere Determine TB LAM Ag (AlereLAM), is a lateral flow assay designed to detect LAM in urine and used as a point-of-care test for active tuberculosis^10–12^.

There is extensive evidence that LAM is found in serum and urine in measurable quantities, established using both chemo-analytical assays and immunoassays^7,8,13–16^; however, only the Alere LAM test is currently in the market for clinical usage. The clinical performance of this test has had a questionable lack of sensitivity; this could be due to a low abundance of LAM in urine in TB patients who have a low bacterial load, particularly for patients who are HIV uninfected. It has been proposed that low LAM could remain undetected due to: first, TB pathology such as kidney dysfunction or dissemination of *M. tuberculosis* to the kidneys^17,18^; second, LAM sequestration in serum and urine, whereby sample pretreatment could enhance the level of detection 100 fold in serum and two to threefold in urine^9,14^; and third, urinary LAM possibly presenting different epitopes than that from LAM in the culture^8,19^.

Studies suggest that refinement of assays may provide improved limits of detection, leading to increased sensitivity and improved performance^20^. To this end, a novel urine based immunoassay, Fujifilm SILVAMP TB LAM (FujiLAM, Fujifilm, Tokyo, Japan) was recently developed and validated^21^. The assay involves the use of two high affinity monoclonal antibodies and a silver amplification step^22^ to intensify the control band and signal bands for LAM in urine, thus increasing the sensitivity of the assay compared to the AlereLAM test. However, the specificity of the FujiLAM test was lower than the AlereLAM test, maybe due to the antibodies used for the test as the presentation of antigenic determinants in urinary LAM in structural organization and increased/reduced multiplicity may have direct consequences on LAM immunoassay. Moreover, the reduced specificity of FujiLAM as indicated by the authors could be partly due to the imperfect reference (culture and Xpert) standard that lacks complete sensitivity. It is limited in its ability to detect TB in patients with HIV infection. Despite the fact that urinary LAM has been validated in several laboratories^9,18,21,23,24^ focusing mainly on HIV infected TB-positive cases, questions remain whether this is applicable to a more general population that includes HIV uninfected TB positive, pediatric TB or extrapulmonary TB patients. Accordingly, the focus of our present study was to test our immunoassay on a well characterized set of urine samples originating from Peru where the incidence of HIV is low. These samples were tested in a simple ELISA format that can be applied in any laboratory. Our assay did not require any urine concentration, involved rigorous sample pretreatment, and utilized monoclonal antibodies (mAb) that are available to us. Overall, in this study, we abolished the concentration step of the sample and instead apply a one-step pretreatment with reduced sample volume and processing time. The test is now more sensitive (LoD increased from ~ 0.1 to 0.05 ng/mL) than earlier report^9^. The data were validated with mass-spectrometry based quantification of LAM in the same samples.

## Methods and materials

### Sample cohort

Anonymized urine samples used in our study were provided by Laboratorio Socios En Salud Sucursal, Peru. The study samples were collected from adult patients with culture confirmed TB (cases; n = 140) and symptomatic children in whom TB was ruled out by a pediatric pulmonologist on the basis of negative bacteriological (i.e., smear and culture) results from sputum or gastric aspirate; chest X-ray, and tuberculin skin testing (controls; n = 20). All samples were collected between 6 and 11 AM in the morning.

Pre anti TB treatment urine samples were collected and EDTA was immediately added to achieve a final concentration of 10 mM. Samples were transported to the lab via cold chain and stored at − 80 °C. Samples were sent to Colorado State University (CSU) in two batches, one set of 60 and a second set of 100 (Table 1). The frozen urine samples were received barcoded and were given arbitrary Colorado State University (CSU) numbers (UP 1–160).

**Table 1:** Clinical Categorization of 160 urine sample set.

Initial cohort (60)	Follow-up cohort (100)
**10 culture negative (children)** All smear (−)	**10 unlikely TB (children)** No smear recorded
**50 culture positive (adult)** 14 smear (−) [28%] 13 smear (+) [26%] 11 smear (++) [22%] 12 smear (+++) [24%]	**90 culture positive (adult)** 26 smear (−) [29%] 4 smear AFB [4%] 29 smear (+) [32%] 23 smear (++) [26%] 8 smear (+++) [9%]

Only 10/60 in the initial cohort and 2/100 in the follow-up cohort were HIV infected. Children are presumed HIV uninfected.

On receipt, the samples were aliquoted into 1 mL aliquots and stored frozen at − 80 °C until use. To prevent contamination, all samples were processed in a biosafety cabinet. All reagents and buffers were sterilized before each use. Samples were collected between May 2015 and February 2018 and analysed in late 2019–March 2020.

Additional urine control samples were obtained from healthy volunteers from a TB non-endemic region and stored frozen at − 80 °C until further use. The control urine (NEU) was spiked with CDC1551 LAM (range 12.5 ng–0.02 ng/mL) to derive an assay standard curve by serially diluting the LAM twofold to obtain a concentration range and compared to the unspiked urine sample which was used as a negative control. A standard curve was routinely extrapolated every time.

### Ethics statement

The study generating the urine samples conformed to the Declaration of Helsinki and was approved by the Ethics Committee of the Peru National Institute of Health and the Institutional Review Board of Harvard Medical School. Written informed consent was provided by adults and guardians, and written assent was obtained from children ages 8 to 14 years. The present research was approved by the CSU Institutional Biosafety Committee (IBC), and the CSU Integrity and Compliance Review Board (IRB) under approval IRB protocol number 09-006B (Lipoarabinomannan Analysis of Urine and Serum).

### LAM for assay standardization

The LAM used in this study was isolated and purified from *Mycobacterium tuberculosis* (Mtb) CDC1551 in large quantities (20–30 mg of LAM isolated from ~ 90-g of wet cells) so that the same standard was used throughout the year for recurring experiments^25^.

### Mouse monoclonal antibodies

CS35 IgG3 and CHCS9-08 IgG3, both mouse monoclonal antibodies raised against *Mycobacterium leprae*, were purified in our laboratory from hybridoma cell lines generated by the fusion of myeloma cells with immunized mouse splenocytes^26^. Briefly, female BALB/c mouse was primed intraperitoneally with whole *M. leprae* cells in presence of complete Freund adjuvant followed by several booster doses of crude LAM extract. Spleen cells were fused with SP20 myeloma cells and hybrid cells were selected and screened by ELISA for the secretion of antibodies reactive to the ethanol extract of delipidated *M. leprae* cells^27^. These antibodies were taken from the Colorado State University repository and no animal experiments was required or performed for this work. Animal experiments conducted about 30 years ago followed IACUC animal protocols and animals received proper care by CSU’s staff veterinarian and laboratory animal care staff following the guidelines and regulations then in place. There was an approved CSU Animal Care and Use Committee protocol at that time and Animal Welfare Assurance number A3572-01 was on file with the Office for Protection from Research Risks at CSU.

### Human IgG1 monoclonal antibody

A194-01 was obtained from New Jersey Medical School, Rutgers University. This antibody was molecularly cloned from a patient diagnosed with pulmonary TB who had been on TB drug therapy for a month before screening the culture supernatant against LAM from Mtb in an immunoassay using a high throughput in vitro B cell culture method. Variable heavy (V_H_) and light (V_L_) chain gene sequences were amplified using RT-PCR followed by nested PCR with different V_H_–V_L_ specific primers, then cloned into an IgG expression vector. The antibody was then expressed by transient transfection of 293 T cells^28^.

### Pretreatment of urine samples

Sample aliquots were thawed on ice before use. A 100 µL aliquot of the urine sample was transferred to a fresh screw-cap tube and treated with Proteinase K (Pro K, Thermo Fisher Scientific) at a final concentration of 200 µg/mL at 55 °C for 30 min followed by inactivation by boiling (100 °C) for 10 min. To check the optimal time required for denaturation of Pro K by boiling the samples, a time course was performed at 0 min, 10 min, 20 min and 30 min. The supernatant obtained after centrifugation at 12,000×*g* for 10 min was directly used for ELISA.

### Immunoassay

Capture ELISA was employed to analyze the clinical samples for presence or absence of LAM as previously described^9^. Polystyrene microplates (Corning Costar) were coated with the capture antibody at a concentration of 10 µg/mL for CS35 or 5 µg/mL for CHCS9-08 in phosphate buffered saline (PBS) and incubated at 4 °C overnight. Urine control samples used for generating a standard curve were spiked with known amounts of LAM and incubated at room temperature for 30 min to allow for the complexation of LAM and protein/s and then stored at -20 °C overnight. Control and clinical samples were pretreated with Pro K and the supernatant used for ELISA. After overnight incubation, the antibody coated plates and the LAM samples were brought to room temperature. The blocking of nonspecific binding sites in the plate wells was done with 1% Bovine Serum Albumin (BSA) in 0.1 M phosphate-buffered saline PBS (blocking buffer) and the plates incubated for 1 h at room temperature. The plates were washed with the blocking buffer before adding the control and the clinical samples to the appropriate wells and incubated for 2 h at room temperature. After washing ten times with 0.1 M PBS/Tw80 (wash buffer), the plates were incubated for 2 h at room temperature with the biotinylated A194-01 at a final concentration of 250 ng/mL. Biotinylation of the antibody was carried out using EZ-Link Sulfo NHS-LC Biotin (Thermofisher Scientific) following the kit protocol and the labeled antibody was desalted on Zeba spin desalting columns 7K MWCO (ThermoFisher Scientific) as per the kit protocol. The plates were washed with wash buffer followed by addition of Streptavidin–Horseradish Peroxidase (R & D Systems) at 1:200 dilution, then incubated at room temperature for 25 min. The plates were washed and Ultra TMB-ELISA chromogenic substrate (ThermoFisher Scientific) added and incubated for at least 30 min and observed for color development. The reaction was stopped by addition of sulphuric acid and the absorbance was read at 450 nm. All controls were run in triplicate and reported as the mean $\pm$ standard deviation. The samples were run in duplicate and plotted against the standard curve. Limits of blank (LoB) and limits of detection (LoD) were generated from the standard curve using Clinical and Laboratory Standards Institute (CLSI) standards^29^.

### Gas chromatography/mass spectrometry analysis for TBSA and d-ara in urine LAM

The method developed has been described in detail^15,16^. Typically, urine samples (1 mL) were subjected to hydrophobic interaction chromatography (HIC) over Octyl Sepharose (OS)-CL 4B. The 40% and 65% n-propanol in 0.1 M NH_4_OAc eluents from the HIC column was divided in two and processed for GC/MS analysis downstream. For tuberculostearic acid (TBSA), the Octyl Sepharose purified LAM from urine was subjected to alkaline hydrolysis to make the corresponding pentafluorobenzyl tuberculostearate derivative. D_2_-palmitic acid (5 ng) was used as an internal standard. The GC/MS analysis of the pentafluorobenzoate ester was carried out using selective ion monitoring program in a negative chemical ionization mode. GC/MS chromatograms with respective peaks were integrated manually (i.e., peak areas were defined manually and integrated areas were generated by the Chromeleon v 7.2 software for the estimation of total TBSA content. The instrument was set to collect data for *m/z* 257.3 (Internal standard) in the range of 5 to 19 min and *m/*z 297.3 after 19 min for TBSA, to collect sufficient data points for low-level mass detection. It was then used to calculate the LAM-equivalent from previously reported formulae^15^. GC/MS analyses were carried out using a Thermo GC-TSQ8000 Evo Triple Quad GC mass spectrometer.

For d-ara estimation, acid hydrolysis (2 M TFA) was carried out to release d-ara and synthesize 1-(α/β-O-(R)-2-octyl)-2,3,5 tri-O-trifluoroactyl-d-arabinofurano/pyranoside. The d-ara derivatives were then analyzed by GC/MS using MS/MS. The ions *m/z* 420.9 (parent ion) to 192.9 (daughter ion), and *m/z* 425.9 (parent ion) to 197.9 (daughter ion) were monitored respectively for d-ara and D-UL-^13^C_5_-arabinose (internal standard; Cambridge Isotope Laboratories Inc., 100 or 10 ng as reported earlier^15^. The amount of LAM-equivalent was calculated using the previously reported formulae (also see equations in the Additional Files). The LoD was set at 2.4 ng, below which peaks appeared as broad humps and could not be quantified. Values below the LoD were designated as LAM negative.

### Statistics

In addition to using a cutoff based on the OD values from a healthy volunteer, the data were analyzed to determine the optimal cutoff with both an ROC-based method (i.e. the value in the data set which gives the largest area under the curve of a receiver operating characteristic curve) and a model-based method (i.e. the point of inflection of a logistic curve, as calculated using coefficients of a logistic model built with the data). Levels of OD_450_ in different smear statuses were evaluated with Kruskal–Wallis tests; if significant, the group means were compared with a Dunn’s test with p-values adjusted for false discovery rate.

The relationship of ranks of OD_450_ values for each smear status to ranks of smear status was also tested using an ordered heterogeneity (OH) statistic^30^. Since there is no inherent numerical measure of distance between levels of smear results, a directional heterogeneity test is used rather than linear regression to examine whether median OD_450_ values increase with smear rank. This statistic is analogous to a one-sided test on whether median OD_450_ values increase as smear category increases. A regression model was built to test the linear relationship of results from the two ELISA tests. Two results were found to be over-influential, creating the appearance of stronger relationship than was true for the majority of the samples. These samples were omitted when building the linear model, resulting in a more conservative estimate of the relationship. A third result was lower than expected in the CS35 OD_450_ value and was omitted in order to meet assumptions for the model.

Rank correlation between the two values was also tested using Kendall’s τ. All samples were included in the calculation of the correlation.

All analyses were conducted in R version 3.6.2^31^.

## Results

### Sample cohort

The 140 adult TB cases had a median age of 32.4 years; child controls had a median age of 8.4. Twelve cases out of 140 were HIV infected and child controls were not tested.

The samples were analyzed in two sets as received (Table 1). The first batch was a set of 60 (Initial cohort) and the second batch was 100 (Follow-up cohort). All were received blind and clinical status was revealed only after analyses was completed.

### Urine pretreatment for LAM ELISA

**Figure 1:**
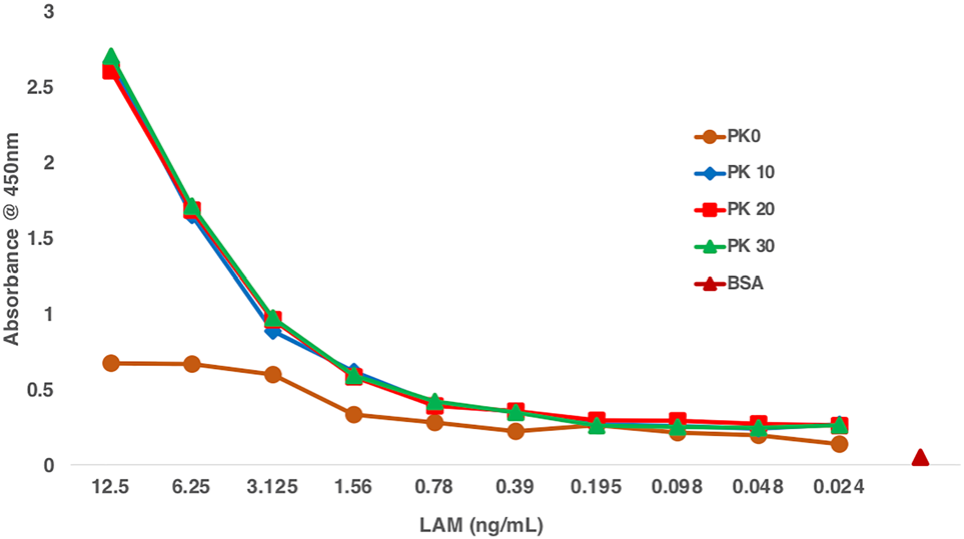
Inactivation of Proteinase K before ELISA, Urine from a healthy volunteer from TB non endemic region was spiked with Mtb (CDC1551) LAM (ranging from 12.5 to 0.02 ng/mL) and pretreated with Pro K (200 ug/mL) to release LAM. Before ELISA, Pro K was inactivated at different time points (0 min to 30 min) to ensure complete inactivation. At 0 min inactivation after pretreatment, the ELISA signal is very weak (brown line), however, with 10 (blue line), 20 (red line) and 30 (green line) min inactivation a proper standard curve is achieved with no difference between the inactivation times.

In the past, the detection of LAM spiked in urine from a healthy volunteer with capture ELISA has consistently been shown to have an LoD at ~ 100 pg/mL after pretreatment with Pro K followed by fivefold concentration prior to ELISA^9^. In the present work, we have optimized the Pro K pretreatment on 100 µL urine and were able to abolish the need for any concentration. We show that the LoD improved two-fold with two sets of antibody pairs (CS35/A194-01 and CHCS9-08/A194-01) to ~ 50 pg/mL, and Pro K inactivation by boiling the samples at 100 °C for 10 min did not inhibit the antibody efficiency (Fig. 1). Thus, these conditions were applied to the processing and analyses of the 160 clinical urine samples.

### Antibody selection

Earlier we reported that using CS35/A194-01 as the antibody pair for capture and detection respectively, after Pro K pretreatment followed by fivefold sample concentration, we achieved a sensitivity of 98% and specificity of 92%^9^. In this study, in addition to CS35, we used CHCS9-08 ms mAb as an alternative capture while maintaining the same detection antibody and found the LoD to be ~ 50 pg/mL.

### ELISA validation on clinical samples

**Figure 2:**
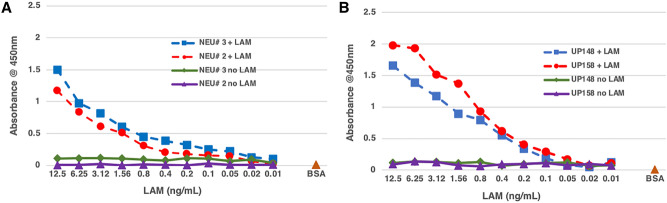
Capture ELISA showing LAM standard curve. Urine from two healthy volunteers (NEU#2, NEU#3) from a TB non endemic region and two clinical samples (UP148, UP158) that were collected from clinically TB negative patients in a TB endemic region were spiked with known amount of LAM and serially diluted two fold (ranging from 12.5 to 0.02 ng/mL) to derive a standard curve and pretreated with Pro K (200 ug/mL). Urine with no LAM but treated with Pro K were used as negative control for background signals. (**A**) Urine from healthy volunteers spiked with LAM show the LoD at ~ 0.05 ng/mL and (**B**) urine from TB negative patients spiked with LAM also show LoD at ~ 0.05 ng/mL. The background signal from urine from one of the healthy volunteers is lower than the signals from the urine from clinical samples with no LAM. This also shows that urine from different sources give varying background levels in an ELISA.

To validate the “no concentration protocol” for ELISA, we used the initial cohort of 60 blinded urine samples from Peru. First, to derive a standard curve and the values for background signals, urine from two healthy volunteers from a TB non-endemic area and two clinically TB negative samples were analyzed. These four samples were spiked with known amounts of LAM (ranging from 12.5 to 0.02 ng/mL) by serially diluting twofold and pretreated with Pro K at 200 ug/mL final concentration before performing the capture ELISA. The 4 samples without LAM but pretreated with Pro K were used as negative control for the background signals. The samples spiked with LAM showed an LoD of approximately 0.05 ng/mL and the unspiked samples showed comparatively negative signals. As expected, we found variations in ELISA OD values among different donors’ urine and TB negative clinical samples (Fig. 2); baseline values were corrected for the absorbance using values of urine with no LAM added but treated with Pro K (negative control for background signals).

**Figure 3:**
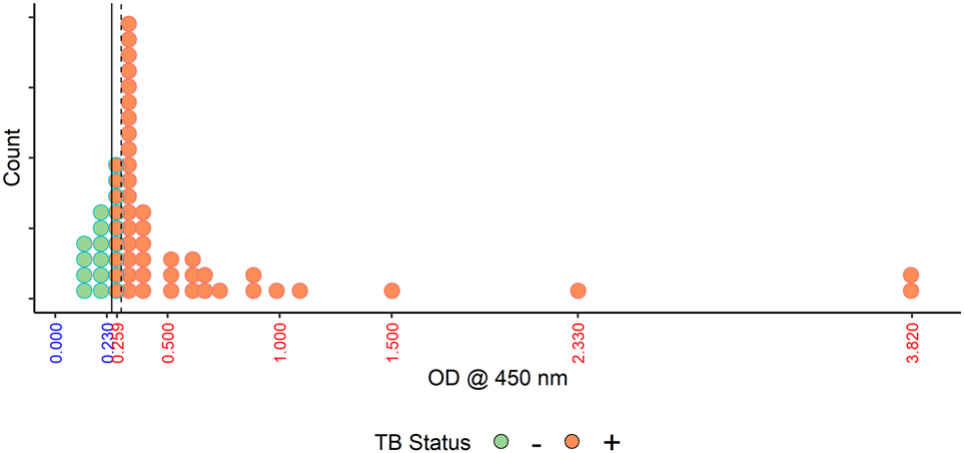
ELISA assay validation on the initial cohort of 60 urine samples. OD values are plotted along the x-axis; each dot represents a single sample. A vertical solid line at 0.252 represents the cutoff value that separates all Non-TB samples from all TB positive samples. A dashed line is placed at the maximum cutoff chosen by bootstrap. The ten samples that would be classified differently with the higher cutoff are outlined in green. Samples in orange dots are all considered positive**.**

The 10/60 samples that were both smear and culture negative were also clearly LAM negative based on the optimal cutoff value. The remaining 50/60 culture confirmed TB cases, 14 of whom were smear negative, were positive for LAM irrespective of their smear gradation (Table 1, Fig. 3; OD_450_ values in Supplementary File Table S1). These sixty samples were evaluated to determine an optimal value of OD_450_ to correctly identify TB positive from non-TB samples. The optimal cutoff value was 0.252 (95% CI (bootstrap): 0.252–0.263). This value perfectly separated the TB positive samples from non-TB samples.

We subsequently analyzed a follow-up set of 100 urine samples. The samples were all blinded as for the initial cohort and processed for capture ELISA using the two separate sets of antibodies (CS35/ biotinylated A194-01 and CHCS9-08 /biotinylated A194-01). When testing, altogether, nine samples out of 100 gave unexpected results in that there was a mismatch of OD_450_ values between the two sets of antibodies used (Table S2). Therefore, a total of 11 samples were retested (UP70, UP74, UP75, UP79, UP94, UP100, UP105, UP140, UP 146, UP156 and UP159). Among these 11 samples there were 9 samples that had ambiguous results the first time and two additional samples (UP75 and UP 159) were retested as a control for ELISA**.** Retested values resulted in 100% accuracy of classification and stronger associations (Supplementary File Fig. S1).

**Figure 4:**
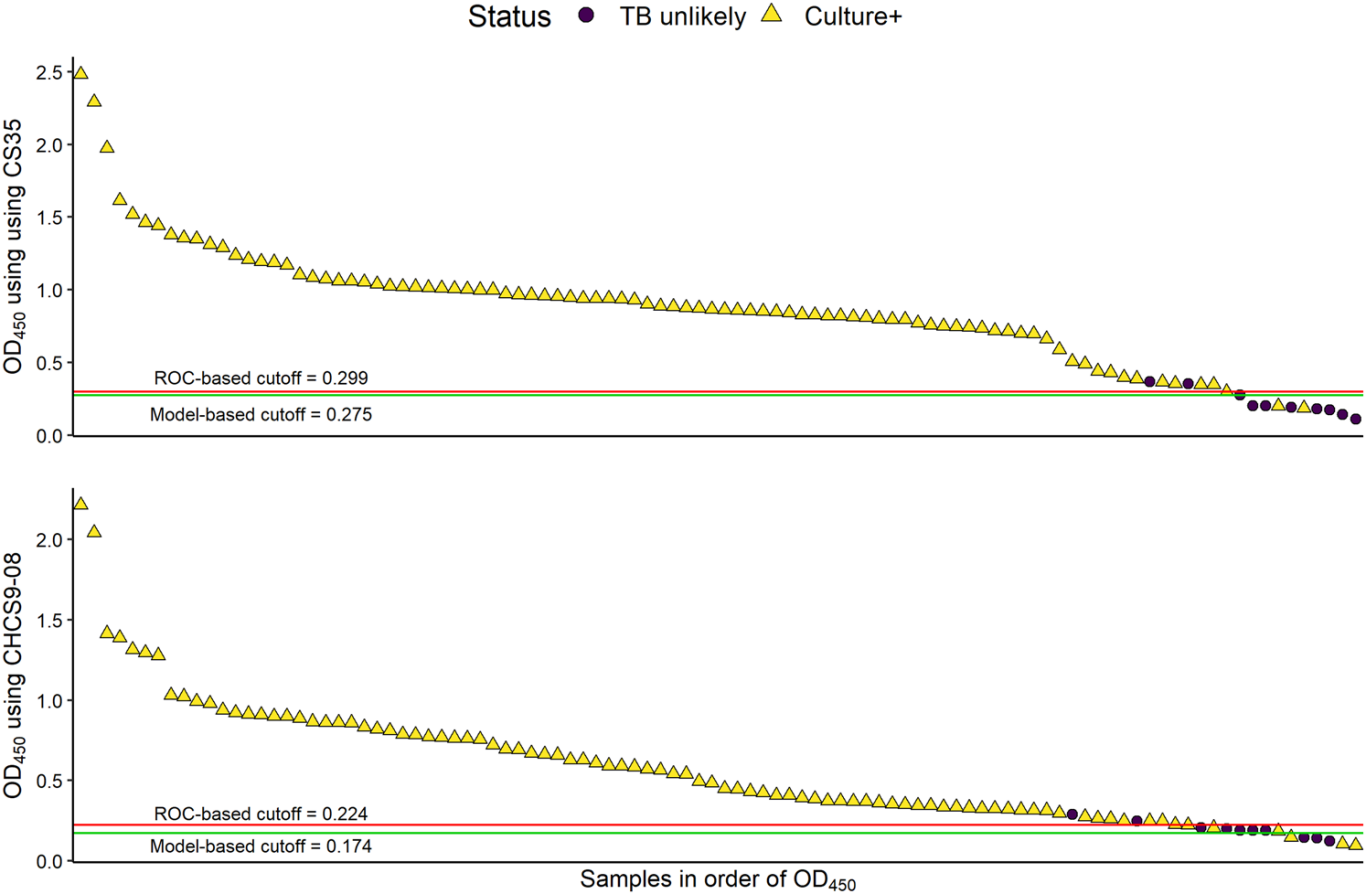
Comparative ELISA on follow-up cohort of 100 urine samples using two sets of antibodies. OD values for 100 samples are listed in descending order, with cutoff values for prediction shown by the horizontal lines. Points below the line were identified as negative, and above the line as positive. Yellow triangles indicate culture positive samples, black dots indicate culture negative samples. 9/100 samples were misidentified perhaps due to experimental error which were later correctly identified (Supplemental File Fig. S1) after reanalysis.

OD values above or at the optimal cutoff value as determined by a logistic model were considered positive for LAM and those below the cutoff value were negative for the test (Fig. 4, Table S2). Using the healthy control as a cutoff, prior to re-testing samples, 95–96% of culture-positive and 80% of culture-negative samples were correctly identified (88% balanced accuracy). An additional culture-negative sample was misclassified using CHCS9-08 prior to re-testing, resulting in 84% balanced accuracy. Because of the small number of negative samples these results may not be as robust as we would like.

### Relationship of ELISA to smear gradation status

Further analyses were conducted using the combined sets. Both ELISA tests showed evidence for differences in OD_450_ comparing those with the smear grade of + versus 3+, although the evidence was weaker for CHCS8-09 (CS35; adjusted p < 0.001; CHCS8-09: adjusted p = 0.045). Culture-negative samples were not included in these analyses.

OD_450_ values from the ELISA tests did not have a linear association with smear values; however, the median LAM value increased with smear rank. This relationship was tested using the ordered heterogeneity (OH) statistic^30^.

The ranks of median ELISA tests using CS35 had a strong relationship to (smear grade) category (OH = 0.97). Because two samples from patients with a high smear grade (2+ and 3+) status had exceptionally high OD_450_ values (Supplementary File Table S2), analyses were re-run without those samples. The relationship remained strong (OH = 0.87). The ranks of median tests using CHCS9-08 also had a strong relationship to smear grade both with (OH = 0.85) and without (OH = 0.83) an outlying result from a patient with a smear grade of 3+ (Supplementary File Fig. S2).

### Comparison of CS35 to CHCS9-08

After 11/100 samples were re-tested, the OD values from the two ELISA tests had a Kendall’s correlation of 0.74, which was only slightly higher than before re-testing. The association indicated by a linear model that excluded the three influential samples was similar (R^2^ = 0.65). On average, OD values from CS35 were 1.5 times higher than those from CHCS9-08 (Supplementary File Fig. S2C).

### LAM quantification of all samples (n = 160) using GC/MS

**Figure 5:**
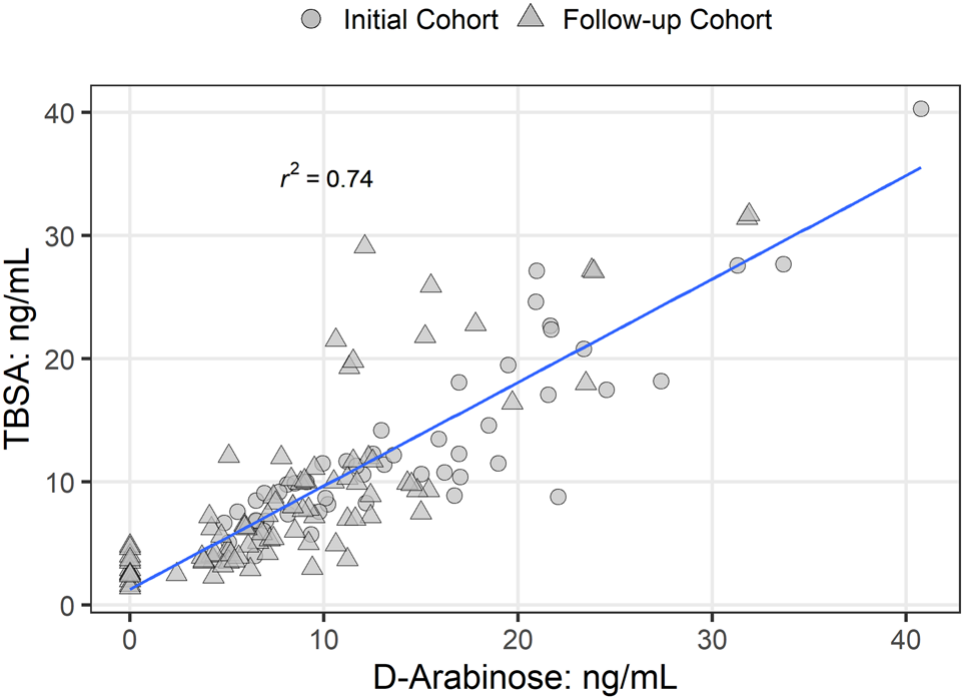
Correlation between d-ara and TBSA by GC/MS analysis in each urine sample analyzed.

LAM from the in vitro grown Mtb is known to be composed of d-arabinose (55–60%), d-mannose (36–40%), and fatty acyls (1–3%; palmitate C:16; tuberculostearate (TBSA) C:19:1)^32,33^. In the context of detecting LAM by GC/MS in urine, d-ara and TBSA were chosen as structural surrogates as the “full-length” LAM is difficult to analyze due to its size and heterogeneity. These monomeric LAM units are unique and mycobacteria specific^34^. The application of HIC, an integral part of our GC/MS protocol, ensures elimination of d-arabinose as a component of similar neutral polysaccharides found in vegetables and food^35,36^. The criteria for calling a sample LAM positive in the GC/MS analysis of TBSA and d-ara is that the same fraction eluting off of the Octyl Sepharose column (40–65%), must have both TBSA and d-ara in amounts that calculate (using set formulae) to be comparable to LAM (i.e. molar ratio of d-ara vs. TBSA). Through this analysis, in this set of 160 samples we found an excellent correlation between d-ara and TBSA analyzed in each sample (Fig. 5). The amount of LAM equivalent was calculated using the previously reported formulae^15^.

**Figure 6:**
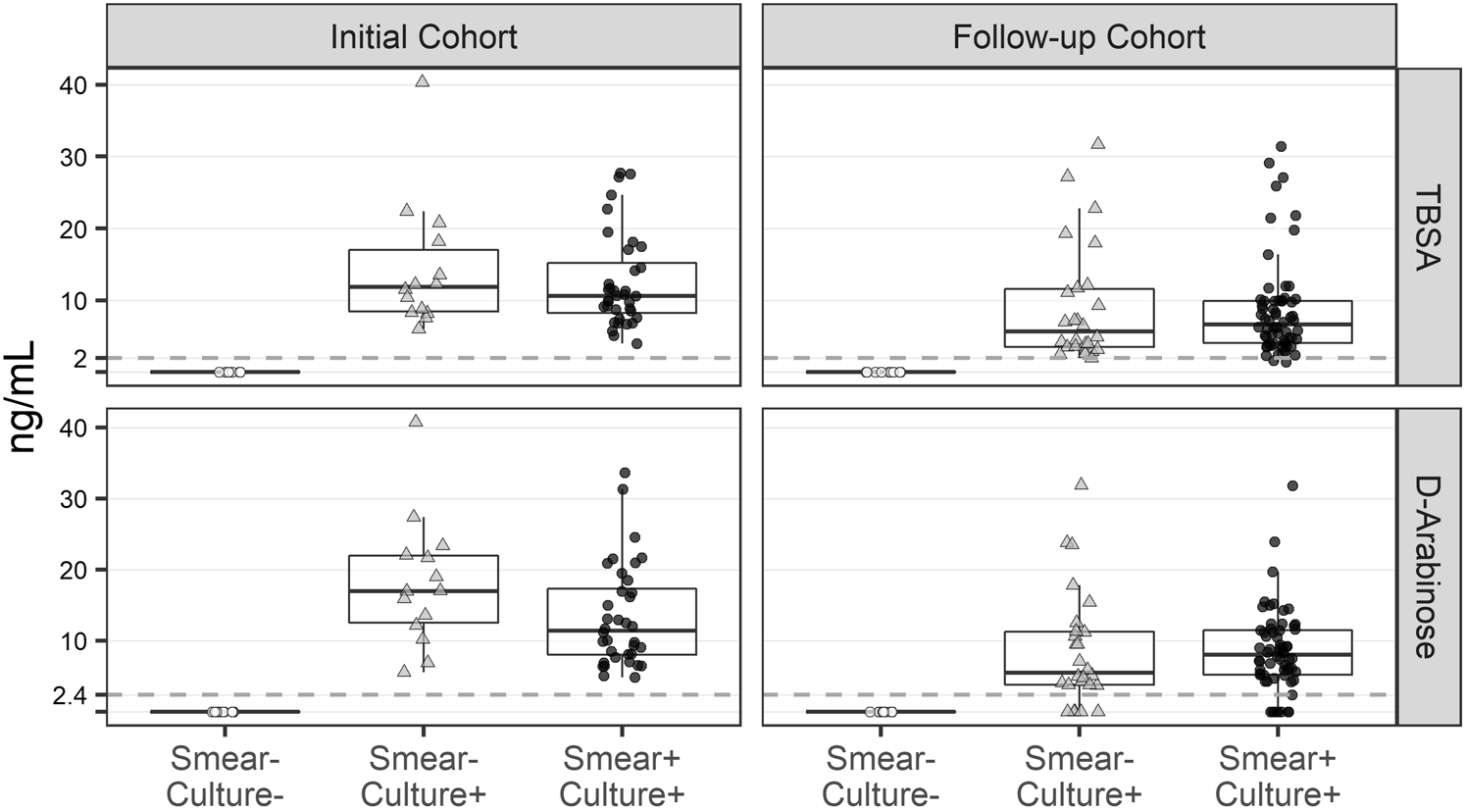
Correlation of smear− and smear+ category with LAM amounts in urine. For TBSA, the LoD is set to be 2 ng/mL below which the peaks are discernable and for d-ara 4 peaks must be present to conclude as positive. LoD for d-ara was found to be < 2.4 ng/mL. Both TBSA and d-ara must calculate out to be in similar amounts (± 5% error for manual integration). Overall, 138 of 140 TB-positive samples has LAM above LoD by TBSA (98.5%) and 127 has LAM by d-ara (90.7%). All 20 clinically TB negative samples had no LAM by either assay (Specificity > 99%).

Furthermore, when we correlated the amount of LAM with smear status, there was not a strong correlation (i.e., samples from patients with a smear grade of 3+ sometimes had lower amounts of LAM than samples from patients with a smear of 1+). However, most importantly, 40/140 urine samples that were smear negative (smear-culture+; Supplementary File Tables S1, S2) had detectable amounts of LAM (Fig. 6). Furthermore, all of these samples were ELISA positive. For all 20 clinically negative samples we could not detect any LAM by either TBSA or d-ara indicating high specificity of these assays.

A representative compilation of GC/MS chromatograms of 20 negatives and 20 positives are provided as Supplemental Files Figs. S3–S10 (raw data). Levels of d-ara and TBSA did not correlate with the ELISA results. A number of the samples had ELISA OD_450_ < 0.51 (42% of samples for CS35 and 64% of samples for CHCS9-08). The top 25% of ranks spread widely, with the top two samples (UP4 and UP45) reaching an OD_450_ of 3.8. However, these two samples had relatively low amounts of d-ara and TBSA by GC/MS. Even when excluding ELISA values > 1.5, the correlation between OD_450_ and GC–MS is low. A compilation of ELISA (with mAb CS35) and LAM quantification with GC/MS are presented in Tables 2 and 3 with a more detailed raw data in Supplementary File Tables S1 and S2.

**Table 2:** Initial cohort of 60 patient’s urine samples from Peru.

Sample ID	Sputum smear	ELISA OD @ 450 nm^a^	TBSA-LAM^c^	D-Ara-LAM^c^	Sample ID	Sputum smear	ELISA OD @ 450 nm^a^	TBSA-LAM^c^	D-Ara-LAM^c^
UP1*	1+	0.866	17.5	24.6	UP31*	2+	0.351	9.8	8.1
UP2	1+	0.2935	10.7	15.0	UP32*	2+	0.635	9.9	8.5
UP3	3+	0.9005	14.2	13.0	UP33	2+	0.323	19.5	19.5
UP4*	3+	3.8175	10.8	16.2	UP34	2+	0.341	18.1	17.0
UP5	1+	0.327	6.8	7.0	UP35	2+	0.987	5.1	5.1
UP6*	3+	0.5905	4.0	6.4	UP36*	3+	1.0905	6.9	6.5
UP7	1+	0.3115	6.8	6.5	UP37*	3+	0.3495	27.7	33.7
UP8	Neg	0.2595	40.3	40.8	UP38	3+	0.31	6.7	4.9
UP9	Neg	0.5005	20.8	23.4	UP39	3+	0.368	10.6	12.0
UP10	Neg	0.3405	18.2	27.4	UP40*	3+	0.414	11.7	11.2
UP11	Neg	0.611	8.2	10.2	UP41	Neg	0.265	22.4	21.7
UP12	Neg	0.3145	11.5	19.0	UP42	2+	1.501	27.6	31.3
UP13	Neg	0.2955	7.6	5.5	UP43	1+	0.5325	11.3	11.7
UP14	Neg	0.259	12.3	16.9	UP44	Neg	0.734	13.5	15.9
UP15	Neg	0.292	10.4	17.0	UP45	3+	3.816	9.2	7.7
UP16	Neg	0.2765	8.3	12.2	UP46	3+	0.662	8.7	10.1
UP17	Neg	0.2645	6.0	6.9	UP47	Neg	0.252	8.8	22.1
UP18	1+	2.332	17.1	21.6	UP48	1+	0.325	11.5	9.9
UP19	1+	0.672	14.6	18.5	UP49	1+	0.395	7.6	9.7
UP20	1+	0.362	8.5	6.5	UP50	Neg	0.292	12.2	13.6
UP21	1+	0.511	5.7	9.3	UP51#	Neg	< Cut-off^b^	< LOD^d^	ND
UP22	1+	0.321	9.1	6.9	UP52#	Neg	< Cut-off^b^	< LOD^d^	ND
UP23	1+	0.378	10.0	9.1	UP53#	Neg	< Cut-off^b^	< LOD^d^	ND
UP24	1+	0.3145	27.2	21.0	UP54#	Neg	< Cut-off^b^	< LOD^d^	ND
UP25*	1+	0.3495	7.4	8.1	UP55#	Neg	< Cut-off^b^	< LOD^d^	ND
UP26	1+	0.3165	12.3	12.5	UP56#	Neg	< Cut-off^b^	< LOD^d^	ND
UP27	1+	0.3835	11.4	13.1	UP57#	Neg	< Cut-off^b^	< LOD^d^	ND
UP28	1+	0.3375	22.7	21.7	UP58#	Neg	< Cut-off^b^	< LOD^d^	ND
UP29	2+	0.263	8.9	16.7	UP59#	Neg	< Cut-off^b^	< LOD^d^	ND
UP30*	2+	0.376	24.6	20.9	UP60#	Neg	< Cut-off^b^	< LOD^d^	ND

50 were sputum culture positive adult TB patients. #10 (UP51-UP60) Culture negative, smear negative pediatric TB negative control from same endemic region.

*Only 10 samples were from HIV infected person.

^a^ELISA was done using CS35 as capture and A194-biotin as detection antibodies and recorded at 450 nM.

^b^ELISA cut-off was set at OD value 0.252.

^c^LAM concentration determined by measurement of tuberculostearic acid (TBSA) and d-Arabinose (D-Ara) and presented in ng/mL.

^d^LOD for TBSA-LAM was 2.0 ng/mL.

**Table 3:** Follow-up cohort of 100 patient’s urine samples from Peru.

CSU ID	Sputum smear	ELISA OD @450 nM^a^	TBSA-LAM^c^	D-Ara-LAM^c^	CSU ID	Sputum smear	ELISA OD@450 nM^a^	TBSA-LAM^c^	D-Ara-LAM^c^
UP61	1+	0.9535	21.8	15.2	UP111	Neg	0.999	3.9	3.7
UP62	1+	0.9415	6.2	4.2	UP112	Neg	1.357	11.1	9.5
UP63*	2+	0.8625	10	10.5	UP113	1+	0.8135	7.7	8.9
UP64	Scanty	1.0245	10.3	11.2	UP114	1+	1.0525	10.1	9
UP65	2+	0.7955	9.9	11.7	UP115	3+	1.102	8.3	7.5
UP66	2+	1.061	12	12.3	UP116	3+	1.015	29.1	12.1
UP67	1+	0.663	4.8	< LOD^d^	UP117	2+	1.196	8	8.4
UP68	3+	1.2915	9.9	8.8	UP118	Neg	1.35	7.2	4.1
UP69	Neg	0.94	27.2	23.8	UP119	2+	0.9295	7.3	7.1
UP70	3+	0.958	10	9.1	UP120	1+	0.9395	5.3	7.2
UP71	1+	1.3125	4	< LOD	UP121	Neg	1.171	12.1	5.1
UP72	2+	0.9375	5	9.2	UP122	2+	0.7505	1.6	< LOD
UP73	Neg	0.349	2.9	< LOD	UP123	2+	1.074	1.4	< LOD
UP74	Neg	0.431	9.3	15.4	UP124	2+	2.29	19.8	11.5
UP75	Neg	0.489	2.6	< LOD	UP125	2+	1.4415	2.9	6.2
UP76	Neg	0.8565	4.2	7.1	UP126	Neg	0.8885	31.7	31.9
UP77	Neg	0.399	2.6	< LOD	UP127	Neg	1.0035	16.4	19.7
UP78	Scanty	0.827	7.5	15	UP128	Neg	1.0395	6.5	5.9
UP79	Neg	0.8215	7	11.2	UP129	Neg	0.964	4	4.2
UP80	1+	0.876	8.8	7.4	UP130	Neg	1.4645	4.4	5.1
UP81	Neg	0.848	22.8	17.8	UP131	Neg	1.084	5.8	6.8
UP82	1+	0.9585	4.6	< LOD	UP132	Neg	1.379	3.5	5.1
UP83	2+	1.0625	3.5	< LOD	UP133	Neg	1.02	4.1	4.4
UP84	1+	0.8295	3.7	11.2	UP134	Neg	0.867	3.2	4.8
UP85	Neg	0.4395	7.2	9.5	UP135	Neg	2.483	4.8	6.1
UP86	3+	1.613	5.1	6.6	UP136	Neg	0.999	4.1	5.2
UP87	Scanty	0.367	3.7	< LOD	UP137	Neg	1.518	3.9	5.6
UP88	1+	1.1895	6	8.5	UP138	Neg	0.972	3.5	3.8
UP89	Neg	0.356	2	< LOD	UP139	Neg	0.9475	2.5	< LOD
UP90	Neg	0.299	11.7	12.5	UP140	Neg	0.7365	3.6	3.8
UP91	Neg	0.348	4.9	10.6	UP141	2+	0.9015	5.4	7.4
UP92	1+	0.389	10.2	8.3	UP142	2+	1.2075	7.2	12.4
UP93	1+	0.8595	3.6	4.3	UP143	1+	1.023	12	7.8
UP94	Neg	0.529	19.3	11.3	UP144#	Neg	< Cut-off^b^	< LOD^d^	< LOD
UP95	Neg	0.5085	4.5	5	UP145#	Neg	< Cut-off	< LOD	< LOD
UP96	1+	0.743	9.3	14.8	UP146#	Neg	< Cut-off	< LOD	< LOD
UP97	2+	0.716	7.8	9.2	UP147	2+	0.967	2.4	< LOD
UP98	1+	0.7	25.9	15.5	UP148#	Neg	< Cut-off	< LOD	< LOD
UP99	1+	0.797	8	8.4	UP149	1+	0.8015	6.3	5.9
UP100	Scanty	0.841	11.7	11.5	UP150#	Neg	< Cut-off	< LOD	< LOD
UP101	2+	1.01	9.9	14.3	UP151	2+	0.698	6.2	6.0
UP102*	Neg	0.883	18	23.5	UP152#	Neg	< Cut-off	< LOD	< LOD
UP103	1+	0.718	2.3	4.3	UP153	2+	0.7575	7	11.6
UP104	3+	1.975	27.1	23.9	UP154#	Neg	< Cut-off	< LOD	< LOD
UP105	2+	0.82	31.4	31.8	UP155	1+	0.589	5.6	4.7
UP106	2+	0.746	8.9	12.4	UP156#	Neg	< Cut-off	< LOD	< LOD
UP107	1+	1.008	2.5	2.4	UP157	1+	1.237	3.6	5.4
UP108	2+	0.853	9.8	14.5	UP158#	Neg	< Cut-off	< LOD	< LOD
UP109	2+	0.809	3	9.4	UP159	1+	0.8735	6.3	5.9
UP110	1+	0.771	21.5	10.6	UP160#	Neg	< Cut-off	< LOD	< LOD

90 were sputum culture positive adult TB patients. #10 Culture negative, smear negative TB negative pediatric control from same endemic region.

*Only 2 (UP63, UP 102) samples were from HIV infected person.

^a^ELISA was done using CS35 as capture and A194-biotin as detection antibodies and recorded at 450 nM.

^b^ELISA cut-off was set at OD value 0.289.

^c^LAM concentration determined by measurement of tuberculostearic acid (TBSA) and d-Arabinose (d-Ara) and presented in ng/mL.

^d^LOD for d-ara-LAM was 2.4 ng/mL and TBSA-LAM was 2.0 ng/mL.

## Discussion

Prior studies have shown that circulating LAM amounts in urine are relatively low in HIV uninfected patients, thereby its detection is below the sensitivity by earlier immunoassays^37,38^. This is in accord with a recent report where large volumes of urine samples were concentrated with nano cage. High affinity copper dye was used as a bait for effectively sequestered LAM away from any potential interfering substances and the analyte concentrated many folds, depending on initial volume used. LAM could be then detected efficiently using enhanced chemiluminescence^20^. We, on the other hand show that urine LAM assay adds to the diagnostic potential for adults who present with TB-related symptoms and a negative sputum AFB smear microscopy. In this study, nearly all case samples were from adults with culture confirmed TB who were HIV uninfected persons who present TB-related symptoms with a negative sputum AFB smear microscopy. In our previous studies, Pro K pretreatment followed by fivefold sample concentration using CS35 and A194-01 as the antibody pair for capture and detection respectively, we achieved a sensitivity of 98% and specificity of 92%. This study cohort of 100 samples from various demographical origin consisted of both HIV infected and HIV uninfected subjects^9^. Equipped with this knowledge, first, we set out to optimize the Pro K pretreatment and observed that after 30 min incubation at 55 °C of LAM spiked urine, followed by inactivation at 100 °C for 10, 20 and 30 min, there was no variation in the absorbance values (Fig. 1) confirming that the antibodies used were stable under these harsh conditions used for pretreatment.

In the present study, first, we tested a small cohort of 60 urine samples (initial cohort) by capture ELISA (Tables 1, 2). Patients at or above the cut-off value were classified as TB LAM positive and below the cut-off value as non-TB. LAM was detected in all 50 samples that were culture and smear positive (36/50) and in 14/50 samples that were culture positive but smear negative. Sputum smear microscopy for acid fast bacilli has a sensitivity of 50–60%^39,40^. Significantly, the 10 clinically TB- (smear and culture) negative urine samples from children had OD_450_ values below the cut-off value and were designated as LAM negative samples (Fig. 3). Classifying patients at or above the cut-off value of 0.252 as TB positive and below the value as non-TB resulted in a 100% sensitivity and specificity. We recognize that the calculated cut-off values maybe too specific to this dataset. Following this, a follow-up cohort of 100 samples were analyzed and for nine samples, there was a mismatch of OD between the two sets of antibodies (CS35/A194-01 and CHCS9-08/A194-01) used. CHCS9-08 is a mouse mAb IgG_3_ subtype and was selected as a capture as it has high affinity to TBLAM^41^, epitope mapping of this antibody is currently under investigation.

Retested values resulted in 100% accuracy of classification and stronger associations (Fig. S1). In general, the urine LAM immunoassay had high diagnostic sensitivity. The sensitivity reported here is not meant to indicate diagnostic accuracy, but rather is pointing out that we have a clear cutoff between the groups. The ELISA improved the overall diagnostic accuracy of sputum AFB testing, however the samples tested were all pulmonary TB. It needs to be further substantiated in extra-pulmonary, paucibacillary TB and pediatric TB**.**

**Figure 7:**
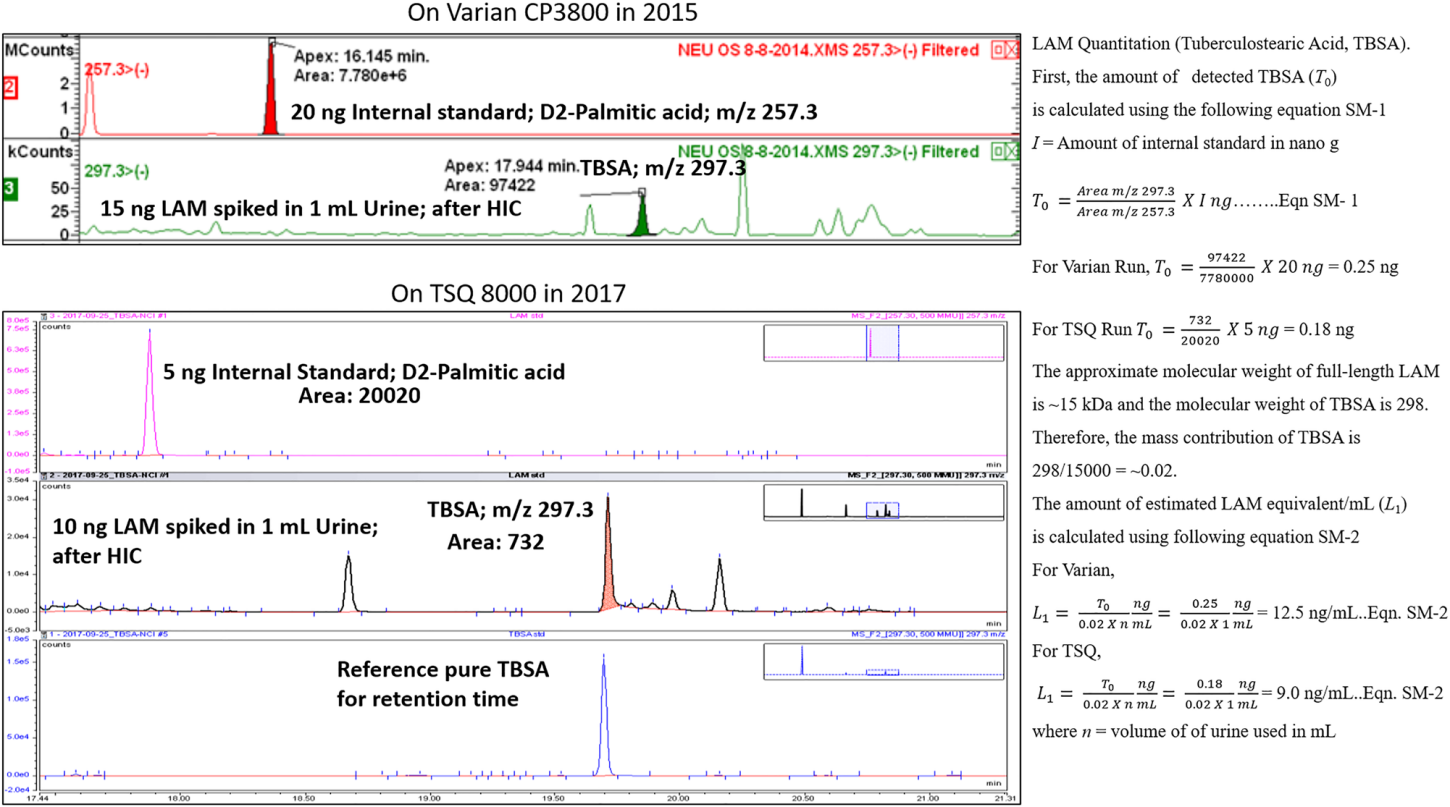
TBSA detection of spiked (non-endemic urine; 1 mL) CDC1551 LAM on Varian CP3800 and TSQ 8000 mass spectrometer in 2015 and 2017 respectively. Out of 15 ng LAM spiked, 12.5 ng was detected on Varian and out of 10 ng LAM spiked, 9.0 ng was detected on TSQ 8000. These spiked samples were subjected to Octyl-Sepharose column chromatography and subsequent pentafluorobenzyl derivative synthesis before GC/MS analyses. D2-Palmitic acid (10 ng and 5 ng respectively) were added to the samples as internal standard before synthesis. Calculations are shown on the right.

The GC/MS analyses of LAM in urine is a robust method and has been validated using over 700 samples to date^9,15,41^. Most of the clinical samples with respective OD_450_ values did not correspond to detected LAM amounts by GC/MS, for instance, very high OD for samples UP4, UP45 and UP135 where LAM amounts by GC/MS are ≤ 10 ng. Whereas one could explain high OD_450_ due to non-specific binding (quite common for clinical samples) or inefficient sample pretreatment. Mtb CDC 1551 LAM has been used to generate the standard curves for both ELISA and GC/MS. We argue that GC/MS methods are true representation of LAM amounts as the quantification of the monomeric units (d-ara 60% and TBSA 1–2% in LAM) reflects their distribution in full-length LAM. The use of known amounts of internal standards such as ^13^C_5_ d-ara and D_2_ palmitic acid makes the quantitation robust. Figure 7 explains the LAM equivalent calculations based on the monomeric unit abundance (TBSA) in samples spiked with known amounts of LAM. Data from two different GC/MS instruments at two different times shows the consistency and reproducibility of the analysis (calculation of d-ara in two representative samples are presented in the Supporting Information Fig S11. In addition, d-ara and TBSA based urine LAM calculations of two representative clinical samples UP81 and UP3 were also included as Supporting Information Figs. S12 and S13 respectively. Based on these calculations, majority of the TB positive samples in this study cohort had ~ 3–40 ng/mL of LAM in urine. The TBLAM amounts in urine is in agreement with published literature that suggested ranges from 1–100 ng/mL^42,43^. On the contrary, in an immunoassay, a mAb will only bind to specific antigenic arabinan epitopes with distinct glycosidic arrangements which is only in the terminal end of LAM. About 30–40% arabinan (α1 → 5 linked) is non immunogenic. Moreover, LAM is structurally heterogenous and the abundance of antigenic epitopes needed for mAbs to bind and response will vary. It is clear that urinary LAM terminal arabinan also varies^19^.

Thus, we anticipate that each urine sample with respect to patient demographic origin may have structurally different LAM. Our present efforts are directed to establish the commonality of urinary LAM to achieve a successful and uniform LAM detection immunoassay.

Nevertheless, the final outcome after applying the two assays does not deter from correctly identifying TB positive vs. TB negative using patients' urine samples. In this study we also show that urine LAM assays have diagnostic potential among HIV uninfected and smear negative patients. Both assays described are detecting LAM with high sensitivity and high specificity, but admittedly are not perfect and not point-of-care. These assays could now move forward to a clinical study encompassing all possible cases of TB. GC/MS is a specialized platform available only in certain laboratories, can be utilized by outsourcing when/if ambiguous results are obtained in immunoassays.

Our study has several strengths and limitations. Primary strengths are the availability of high quality, properly characterized clinical samples, in-house purified ELISA reagents, and an antibody independent validation method for clear assignment of presence or absence of LAM in clinical samples. The ELISA is reproducible, needs no sophisticated instrumentation and can be adapted in any clinical setting, with readouts obtained in 24 h.

Limitations include unavailability of a large set of TB negative samples from the same study group. The TB negative samples in our study were from children. We did not have samples from symptomatic adults in whom TB was ruled out. Therefore, we estimated specificity from samples collected from children with signs and symptoms of TB and a household contact, but in whom TB ruled out by a pediatric pulmonologist following a careful work-up, which included chest X-ray, sputum smear and culture and tuberculin skin testing. Assessing clear demarcation between TB positive vs. TB negative when bacterial load is low or at borderline levels as seen in samples is difficult (e.g*.* UP146, UP156 (Table S2) and so on). In some cases, ELISA needed to be repeated to obtain unambiguous data; more importantly, the sample pretreatment with Pro K needs further optimization (currently pursued in the laboratory). The most important aspect of any immunoassay development is the high affinity capture antibody. We strongly feel this is the area that needs to be strengthened for future development of a TB specific sensitive assay. We recently observed that our TB ELISAs do not work with samples from CF patients with NTM infection for reasons unexplained^41^. LAM being extremely heterogeneous with many structural intricacies, there could be multiple epitopes that will eventually direct its binding capacity. We are already seeing some evidence in this aspect^8,19,41^. In addition, all samples tested to date have been through at least three freeze thaw cycles and the condition of clinical samples may lead to variable ELISA performance from time to time. Although LAM is thermo stable and freeze thaw cycles should not affect the antigenicity of LAM, however, there could be adverse effects (contamination, complexation etc.) on the biological matrix (urine). We hope future research will better inform these areas of uncertainty to enable a more comprehensive and evidence based method of diagnosing pulmonary TB.

## Supplementary Information

Supplementary Figures

Supplementary Tables
