# Supplementary material for: Urine lipoarabinomannan in HIV uninfected, smear negative, symptomatic TB patients: effective sample pretreatment for a sensitive immunoassay and mass spectrometry
Source: Sci Rep. 2021 Feb 3;11:2922. doi: 10.1038/s41598-021-82445-4 (PMC7859189; doi:10.1038/s41598-021-82445-4)
Supplement: Supplementary file 1 — Supplementary Figures [file 41598_2021_82445_MOESM1_ESM.pdf]

Supporting Information

Urine Lipoarabinomannan in HIV uninfected, smear negative, symptomatic TB patients: Effective sample pretreatment for a sensitive Immunoassay and Mass Spectrometry

Anita G. Amin[1], Prithwiraj De[1], Barbara Graham[1], Roger Calderon[2], Molly F. Franke[3] and Delphi Chatterjee[1*]

[1]Mycobacteria Research Laboratory, Department of Microbiology, Immunology and Pathology, Colorado State University, Fort Collins, CO 80523, USA
[2]Laboratorio de Socios En Salud, Sucursal Peru, Av. Chimpu Ocllo N 998 Urb Res., Lucyana, Carabayllo, Peru
[3]Department of Global Health and Social Medicine, Harvard Medical School, Boston, MA 02115, USA

Corresponding Author

E.Mail: delphi.chatterjee@colostate.edu; Tel: +1 970 491 7495

CoAuthors E.Mail:

anita.amin@colostate.edu
prithwiraj.de@colostate.edu
barb.graham@colostate.edu
rcalderon_ses@pih.org
molly_franke@hms.harvard.edu

Keywords Lipoarabinomannan, immunoassay, tuberculosis, diagnosis, urine pretreatment

Table of Contents:

Page	Figure	Description
S-3:	Fig S1	Comparative ELISA on 160 urine samples using two sets of antibodies. OD_{450} values are listed in descending order.
S-4	Fig S2A	ELISA correlate to smear (BK) gradation status with mAb CS35
	Fig S2B	ELISA correlate to smear (BK) gradation status with mAb CHCS9-08
S-5	Fig S2C	ELISA Correlation between CS35 and CHCS9-08. Samples were run at different times.
S-6-S-13	Fig S3- S10	Representative GC/MS Chromatograms of D-Arabinose and TBSA Analysis of 20/160 urine samples includes both culture positive and culture negative samples.
S-14	Fig S11	GC-Chromatogram and explanation of D-Arabinose based LAM quantity calculation
	Fig S12	Description of LAM quantitation from D-Arabinose and TBSA for clinical sample UP81
S-15	Fig S13	Description of LAM quantitation from D-Arabinose and TBSA for clinical sample UP3

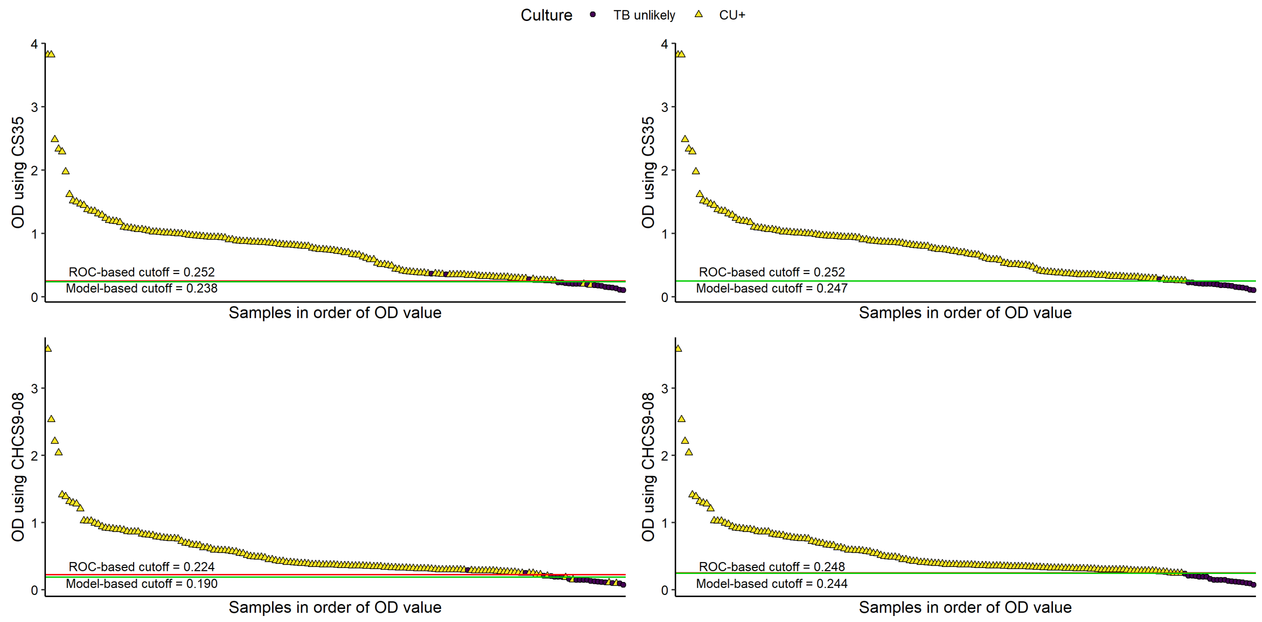

Fig.S1 OD_{450} values for 160 samples are listed in descending order, with cutoff values for prediction shown by the horizontal lines. Points below the line were identified as negative, and above the line as positive. Yellow triangles indicate culture-positive samples, black dots indicate culture-negative samples. Eleven samples were rerun on ELISA assuming misidentification was due to technical error (Left panel). These all got corrected after reanalysis (Right panel).

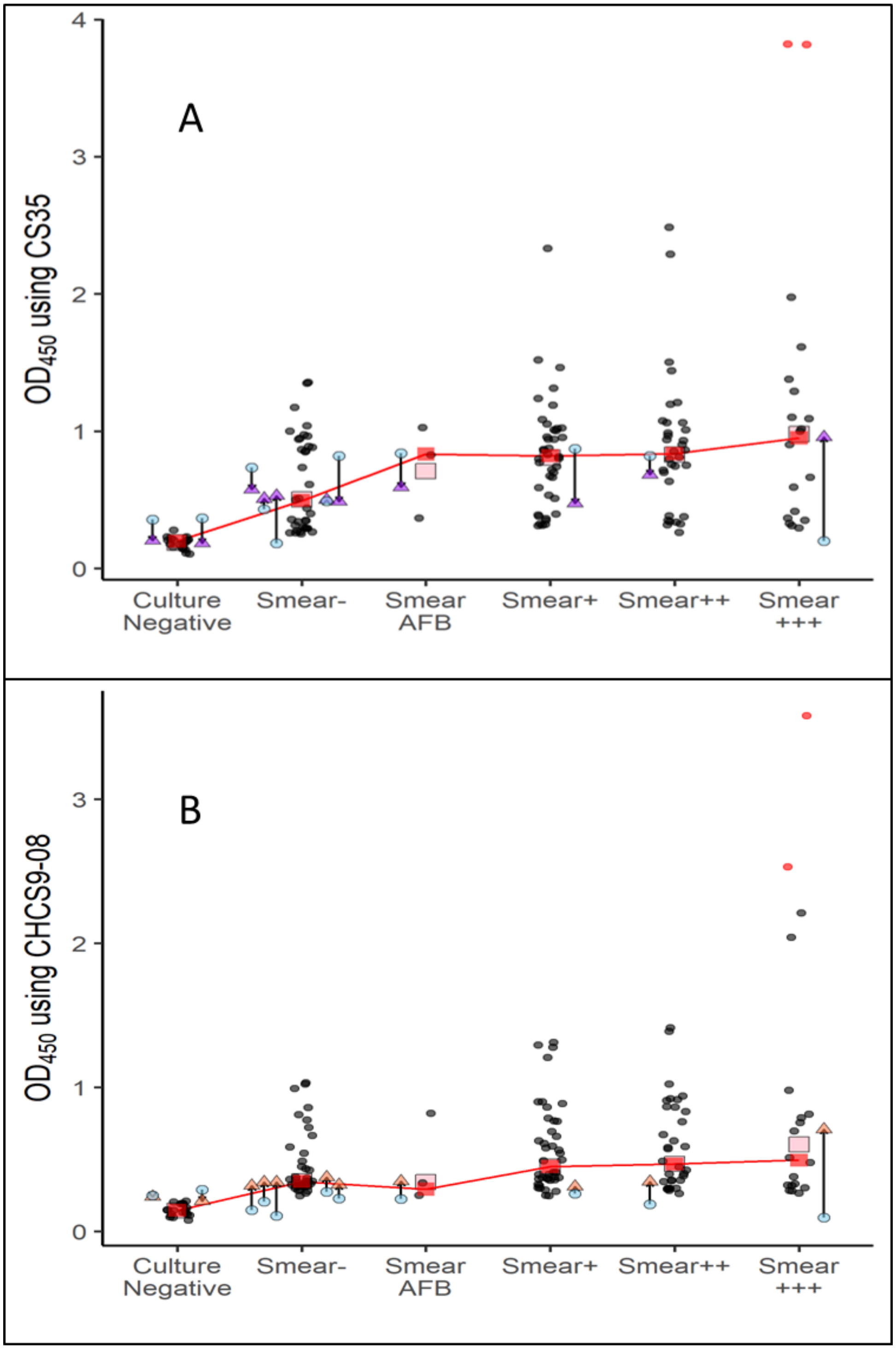

Fig. S2A and S2B. OD_{450} values for CS35 and CSCH9-08 in each of the smear categories for combined 160 urine samples. Red points in the smear grade +++ category was excluded when calculating the OH statistic. Eleven samples were retested. Blue circles show the original values; arrows point to the triangles showing the retested values. Red squares show the median for each category; pink squares are the median after samples were retested.

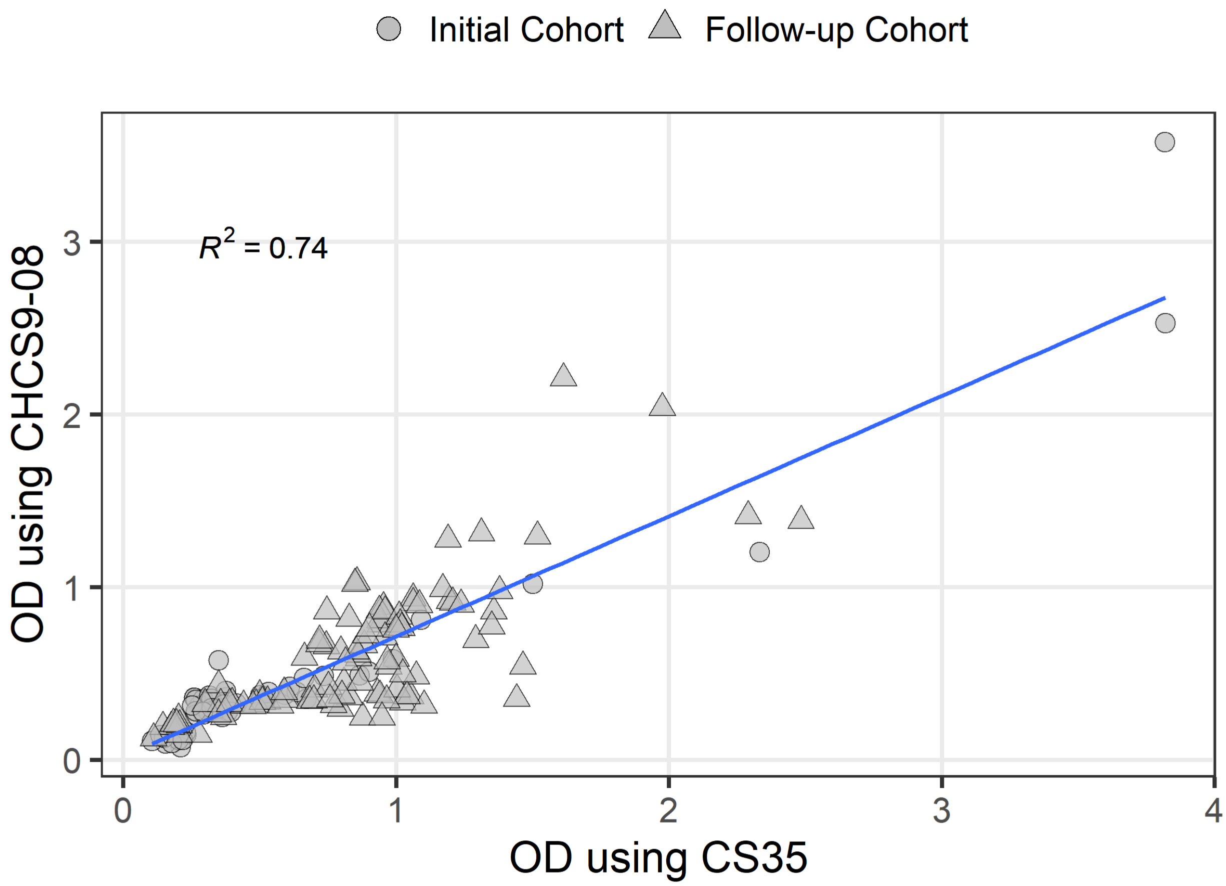

Fig. S2C. OD_{450} values for ELISA tests using the two antibodies are shown with the CS35 antibody on the x-axis, and the CHCS9-08 antibody on the y-axis. A regression line is shown in blue; the coefficient of determination was 0.74. Exclusion of three influential samples resulted in $R^2 = 0.65$."

Supporting info fig S3-S10

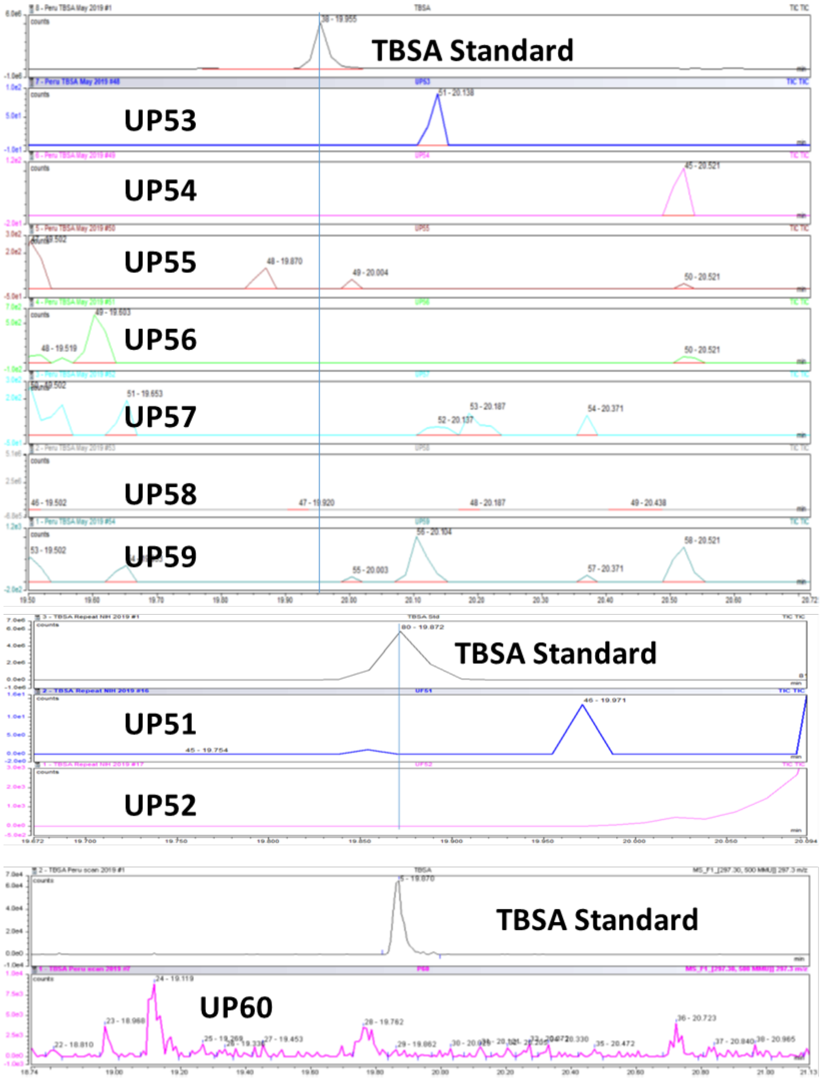

Fig S3: The TBSA GC/MS chromatograms of TB-Culture-Neg Samples UP51-UP60. Peaks appear to be missing at the respective retention times shown with Standard TBSA (First, ninth and twelfth panels). Consequently, these samples were designated as TBSA-LAM Negative.

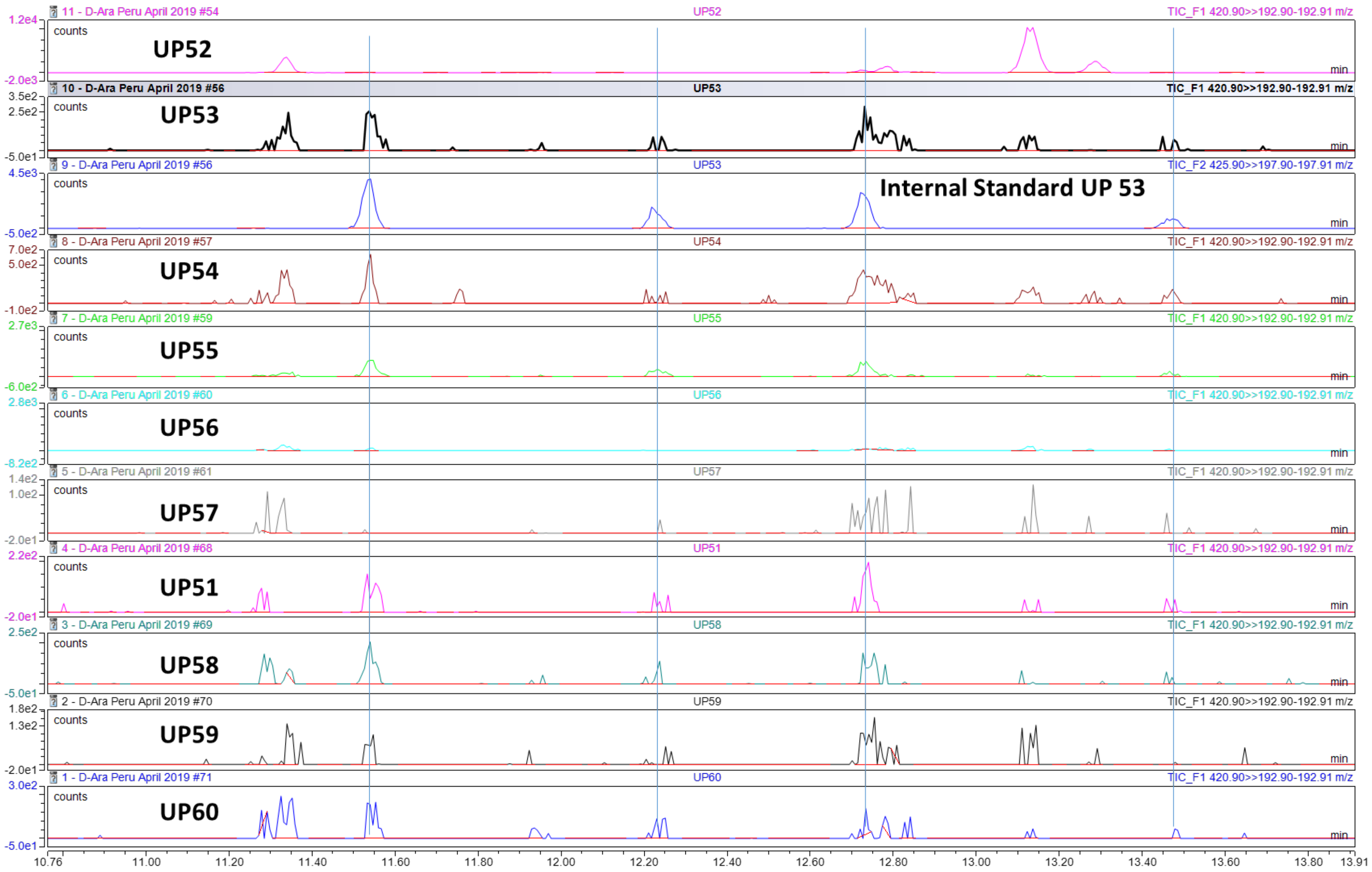

Fig S4: The D-Arabinose GC/MS chromatograms of TB-Culture-Neg Samples UP51-UP60. Peaks appear to be missing or noise-like at the respective retention times shown with internal standard of UP 53 (third panel). Consequently, these samples were designated as D-Ara-LAM Negative.

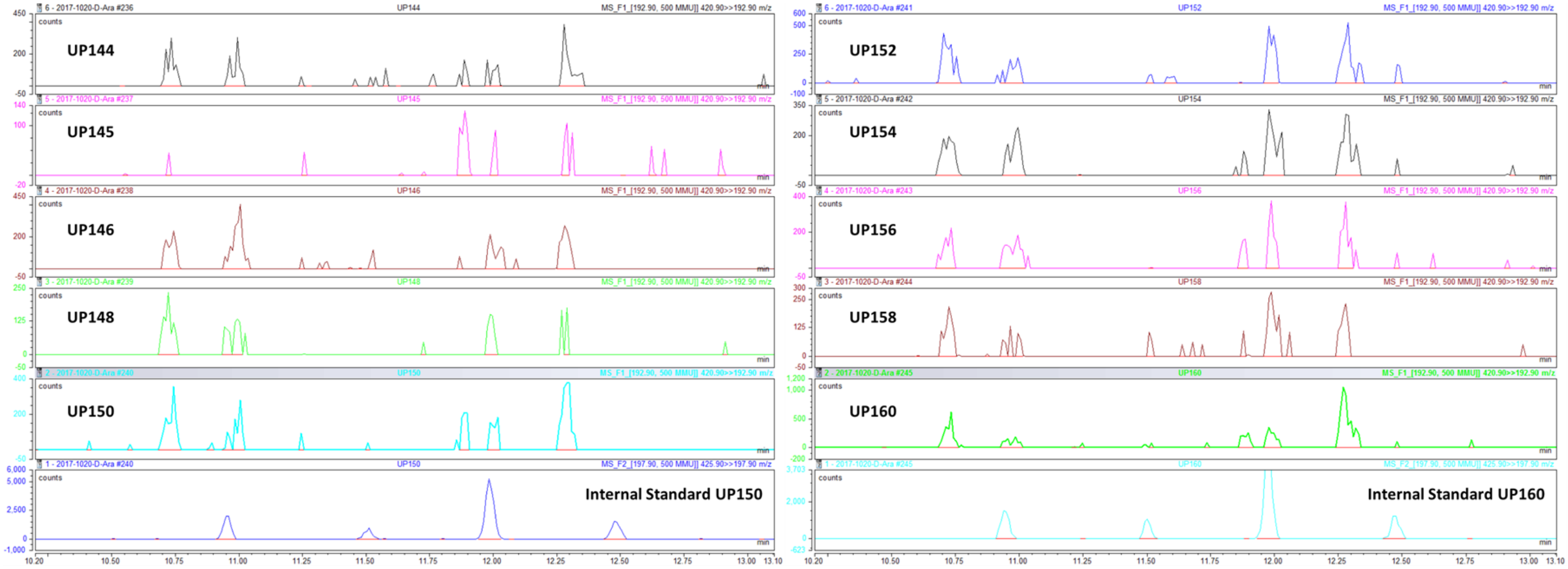

Fig S5: The D-Arabinose GC/MS chromatograms of TB-Culture-Neg Samples UP144-146, UP148, UP 150, UP152, UP154, UP 156, UP 158 and UP160. Peaks appear to be missing or noise-like at the respective retention times shown with internal standard of UP150 (bottom panel left) and UP160 (Bottom panel right). Consequently, these samples were designated as D-Ara-LAM Negative.

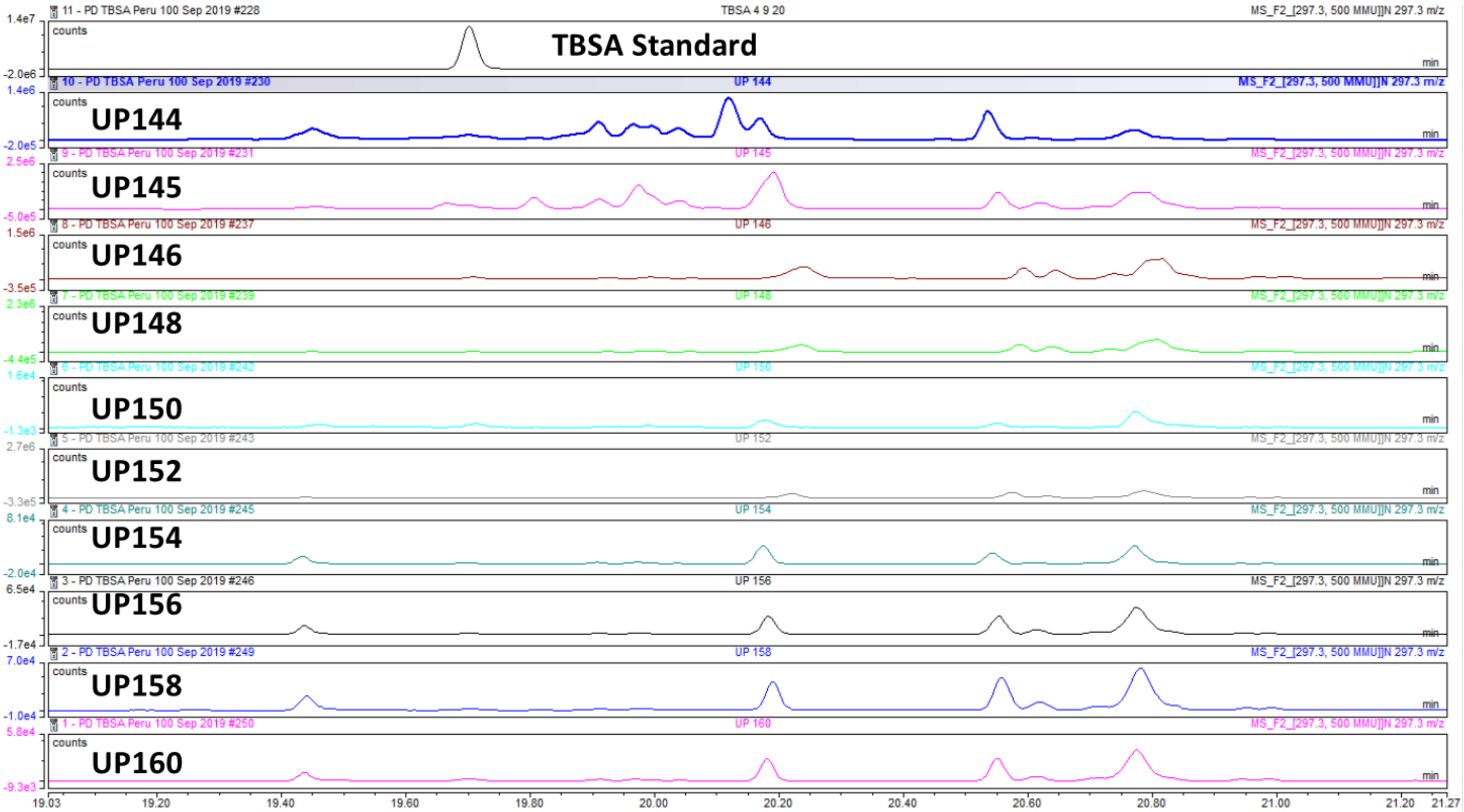

Fig S6: The TBSA GC/MS chromatograms of TB-Culture-Neg Samples UP144-146, UP148, UP 150, UP152, UP154, UP 156, UP 158 and UP160. Peaks appear to be missing or noise-like at the respective retention times shown with Standard TBSA (First panel). Consequently, these samples were designated as TBSA-LAM Negative.

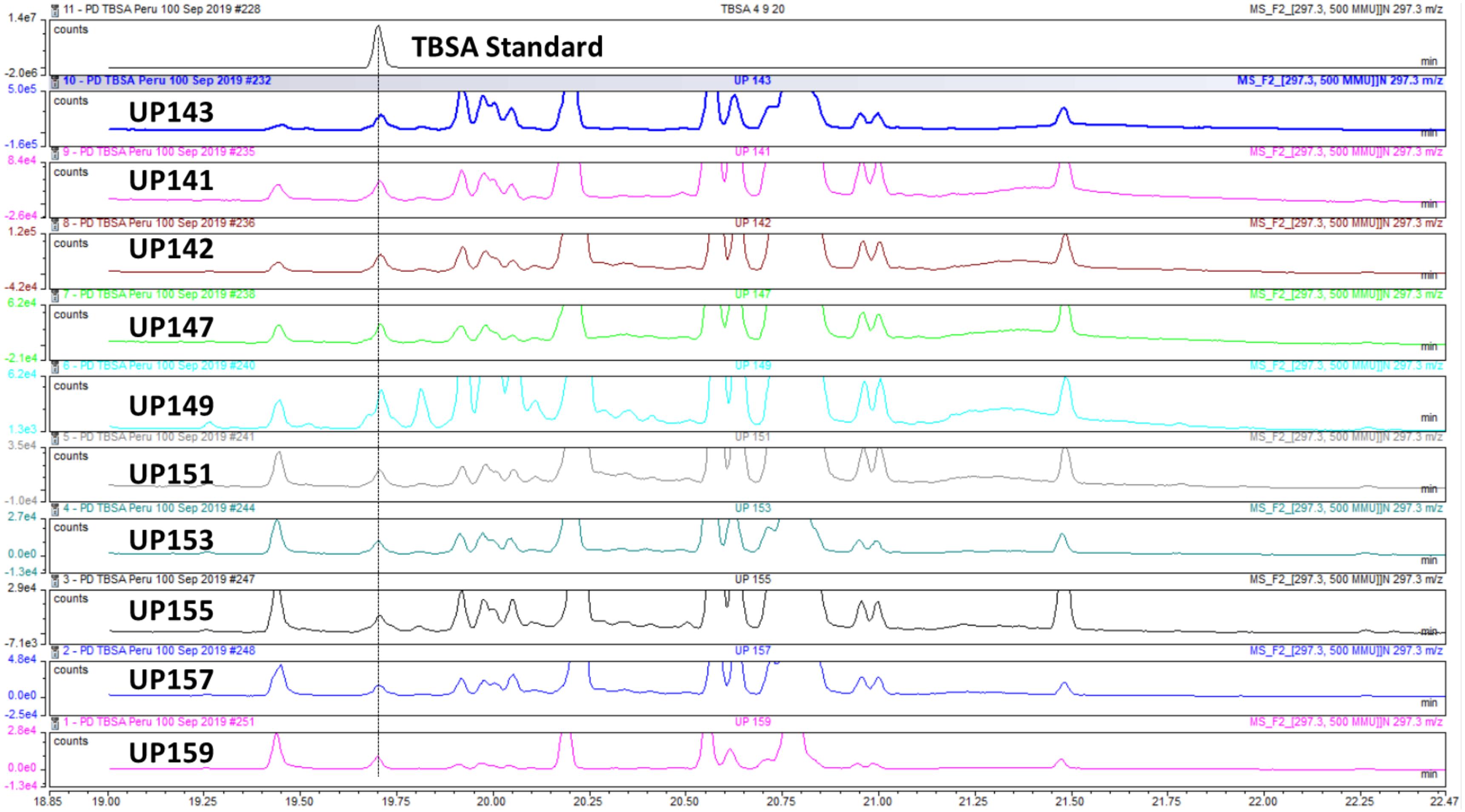

Fig S7: The TBSA GC/MS chromatograms of TB-Culture-positive Samples UP141-143, UP147, UP 149, UP151, UP153, UP 155, UP 157 and UP159. Peaks appear to be nicely aligned at the respective retention times shown with Standard TBSA (First panel). Consequently, these samples were designated as TBSA-LAM positive.

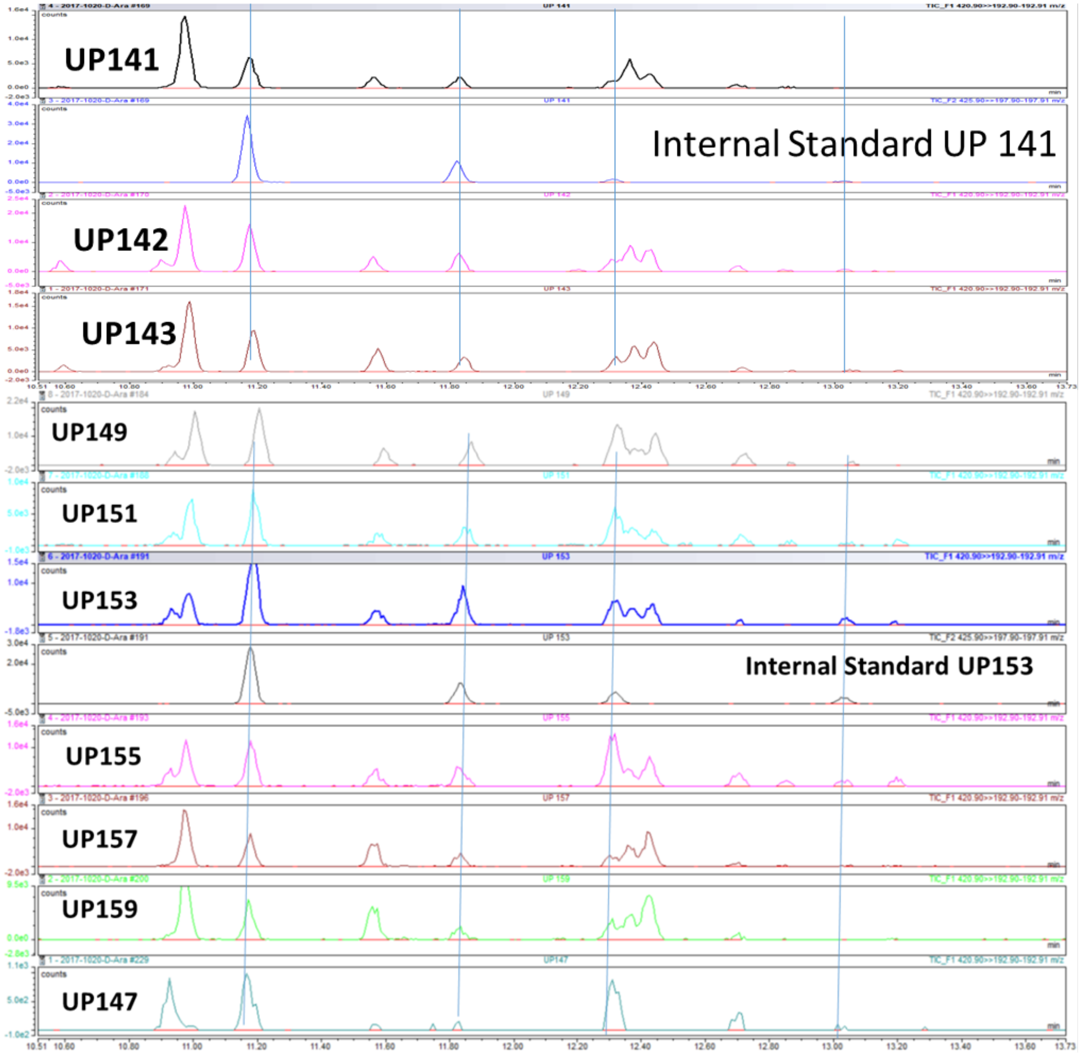

Fig S8: The D-Arabinose GC/MS chromatograms of TB-Culture-Positive Samples UP141-143, UP147, UP 149, UP151, UP153, UP 155, UP 157 and UP159. Peaks appear to be nicely aligned (except UP147, bottom panel) at the respective retention times shown with internal Standard $^{13}C_5$-D-Ara (Second panel (IS UP141) and eighth panel (IS UP153)). Consequently, these samples were designated as D-Ara-LAM positive (UP147 was designated as not detected).

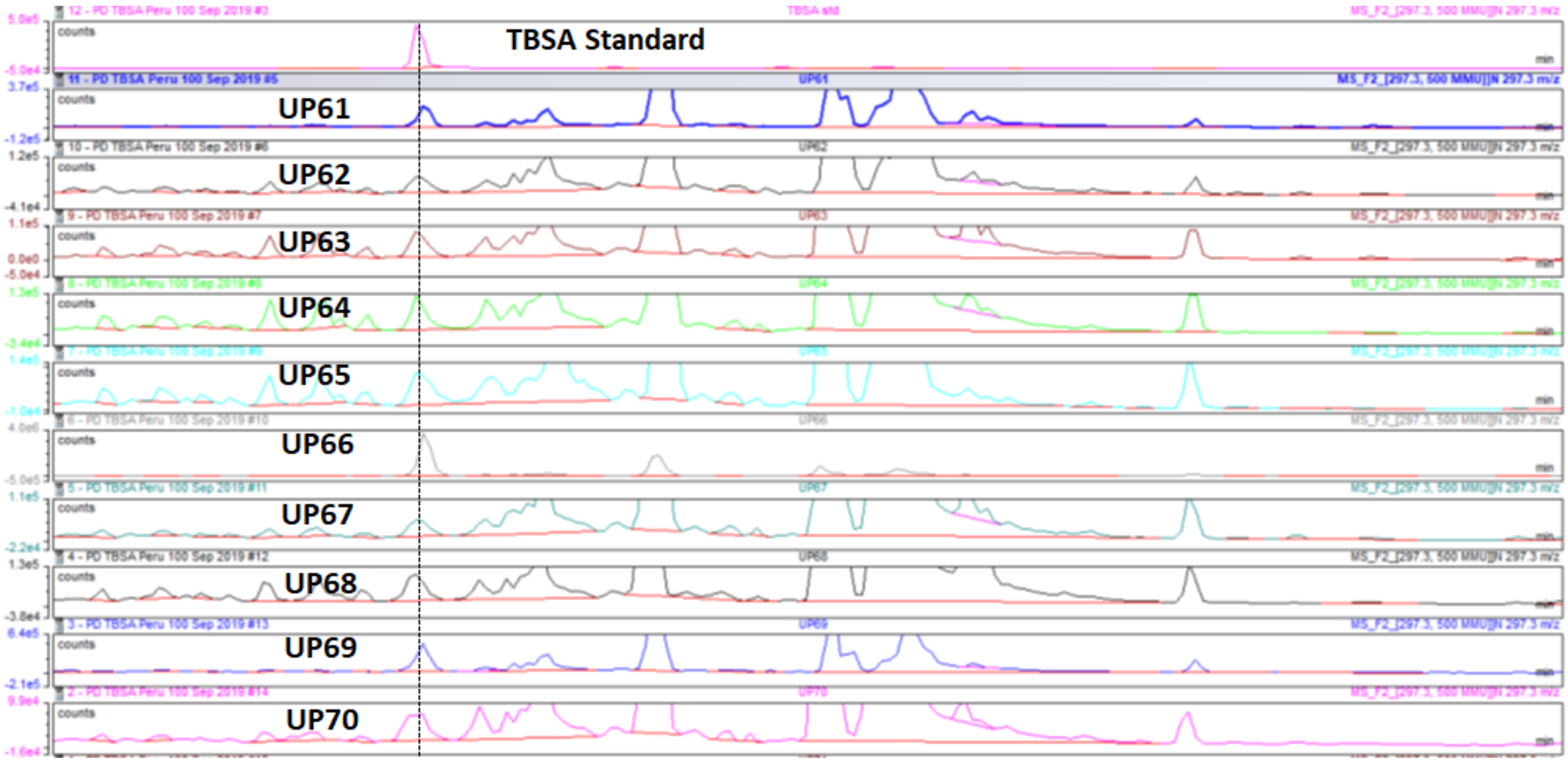

Fig S9: The TBSA GC/MS chromatograms of TB-Culture-positive Samples UP61-70. Peaks appear to be nicely aligned at the respective retention times shown with Standard TBSA (First panel). Consequently, these samples were designated as TBSA-LAM positive.

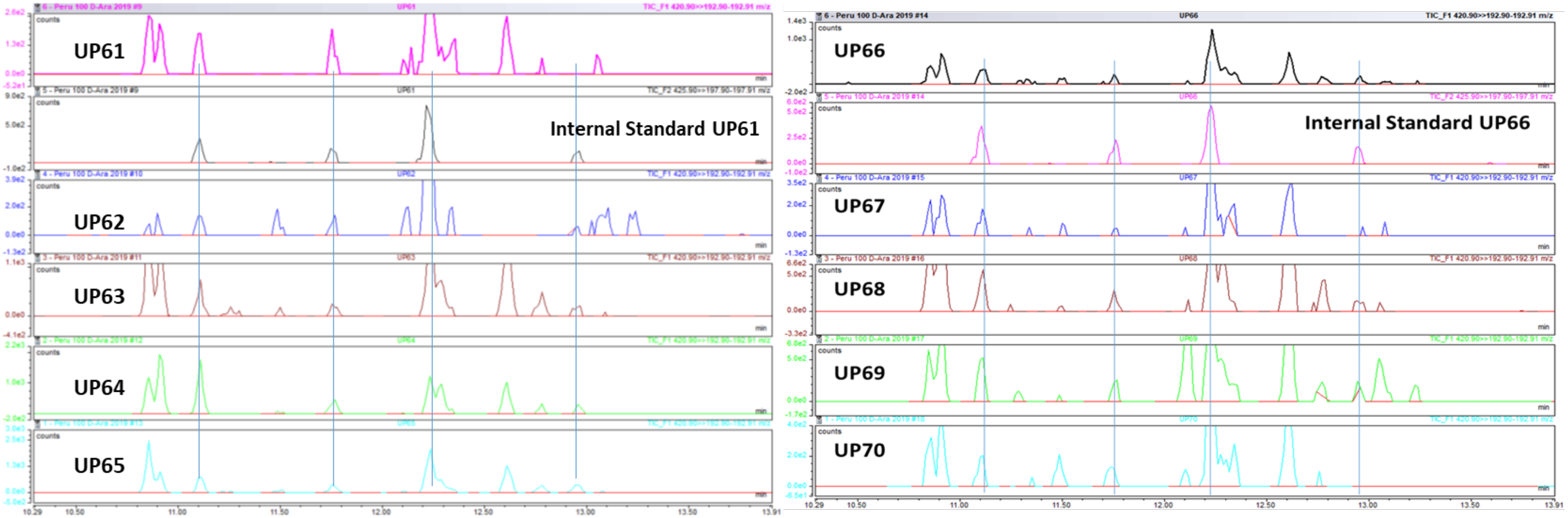

Fig S10: The D-Arabinose GC/MS chromatograms of TB-Culture-Positive Samples UP61-70. Peaks appear to be nicely aligned at the respective retention times shown with internal Standard $^{13}C_5$-D-Ara (Second panel Left (IS UP61) and second panel right (IS UP66)). Consequently, these samples were designated as D-Ara-LAM positive.

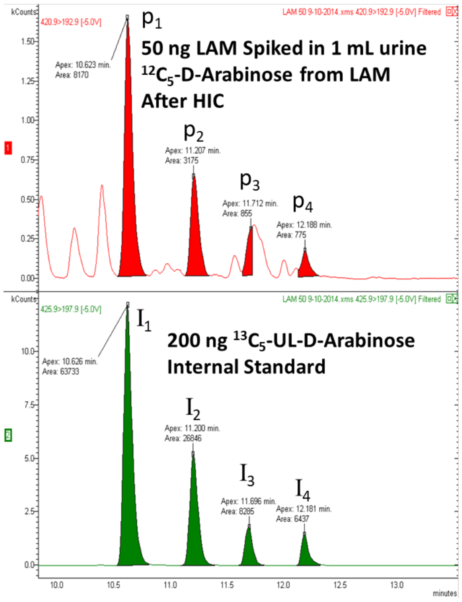

LAM Quantitation (D-arabinose). For quantitation, theoretically the ratio of internal standard to sample arabinose can be used for any one of the four isomeric peaks of the $^{13}C_5$-D-arabinose/$^{12}C_5$-D-arabinose since both the stable isotope labeled internal standard and the unlabeled D-arabinose in the sample will give identical ratios of the α and β anomers of the pyranosyl and furanosyl ring forms for a given sample (the ratio may vary between samples). As it turned out, that all four peaks were clean of contaminants as shown by a consistency of the ^{12}C-D-arabinose/^{13}C-D-arabinose (200 ng used) and therefore all four peaks were used in the calculation. This lead to Eqn. SM-1to determine the amount of arabinose in the tube:

$$A_0 = \frac{1}{4}\left(\frac{p_1}{I_1}+\frac{p_2}{I_2}+\frac{p_3}{I_3}+\frac{p_4}{I_4}\right) X\ 200\ ng = \frac{1}{4}\left(\frac{8170}{63733}+\frac{3175}{26846}+\frac{855}{8285}+\frac{775}{6437}\right) X\ 200\ ng = 23.5 \text{ ng} \quad \text{[Eqn. SM-1]}$$

where A_0 is the amount of D-arabinose in the tube in nanograms. (p_1, p_2, p_3, p_4) are the peak areas of the ion from $^{12}C_5$-D-arabinose and (I_1, I_2, I_3, I_4) are the areas of the ion from the internal standard $^{13}C_5$-D-arabinose. The amount of LAM per milliliter (L_1; n = volume of urine used in mL) was then calculated by Eqn. SM-2, after consideration that the mass contribution of D-arabinose to full-length LAM is 60% (5).

$$L_1 = \frac{A_0}{0.6}/(n) \text{ ng/mL} = \frac{23.5}{0.6}/(1) = 39.2 \text{ ng/mL} \qquad \text{[Eqn. SM-2]}$$

Fig S11: Urinary LAM was spiked with 50 ng CDC1551 LAM. Subsequent octyl-sepharose column chromatography and D-Arabinose analysis by GC/MS detected 39.2 ng. Isotopically labeled $^{13}C_5$-D-Arabinose (200 ng) was used as the internal standard to detect natural $^{12}C_5$-D-Arabinose before chemical derivatization and GC/MS analysis. Calculations are shown on the right.

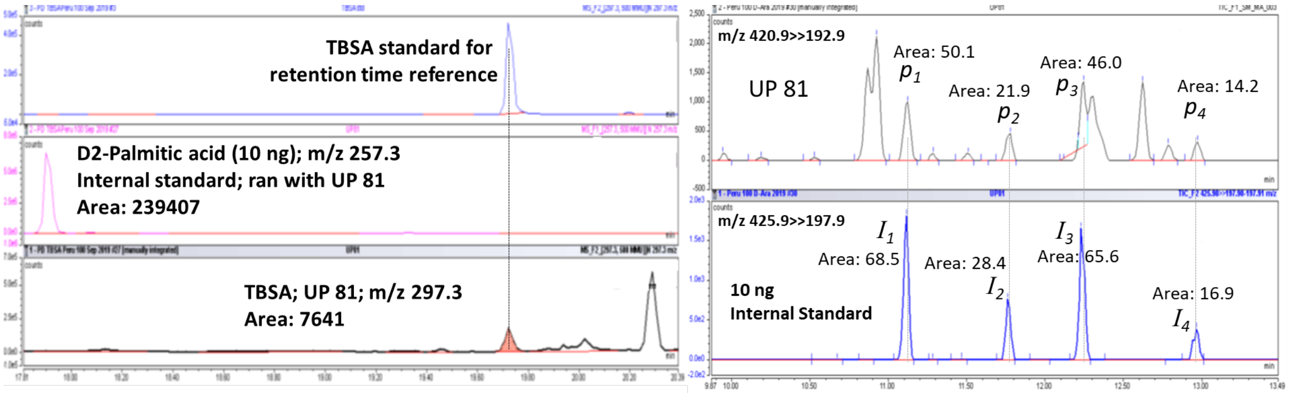

The amount of detected TBSA (T_0); I = Amount of internal standard ; 10 nano g

$$T_0 = \frac{Area\ m/z\ 297.3}{Area\ m/z\ 257.3}\ X\ I\ ng = \frac{7641}{239407}\ X\ 10\ ng = 0.32 \text{ ng}$$

The mass contribution of TBSA is 298/15000 = ~0.02.

The amount of estimated LAM equivalent/mL (L_1)

For UP81, n = volume of urine; 0.7 mL

$$L_1 = \frac{T_0}{0.02\ X\ n}\frac{ng}{mL} = \frac{0.32}{0.02\ X\ 0.7}\frac{ng}{mL} = \mathbf{22.8\ ng/mL}$$

Total D-Arabinose,

$$A_0 = \frac{1}{4}\left(\frac{p_1}{I_1}+\frac{p_2}{I_2}+\frac{p_3}{I_3}+\frac{p_4}{I_4}\right) X\ 10\ ng = \frac{1}{4}\left(\frac{50.1}{68.5}+\frac{21.9}{28.4}+\frac{46.0}{65.6}+\frac{14.2}{16.9}\right) X\ 10\ ng = 7.5 \text{ ng}$$

The amount of LAM per milliliter (L_1; n = volume of urine used in mL; here 0.7 mL)

$$L_1 = \frac{A_0}{0.6}/(n) \text{ ng/mL} = \frac{7.5}{0.6}/(0.7) = \mathbf{17.8\ ng/mL}$$

Fig S12: Detection of TBSA and D-Arabinose for sample UP 81 as a representative. Respective internal standards (10 ng each) were used for each assay. Any loss during the process is not accounted for.

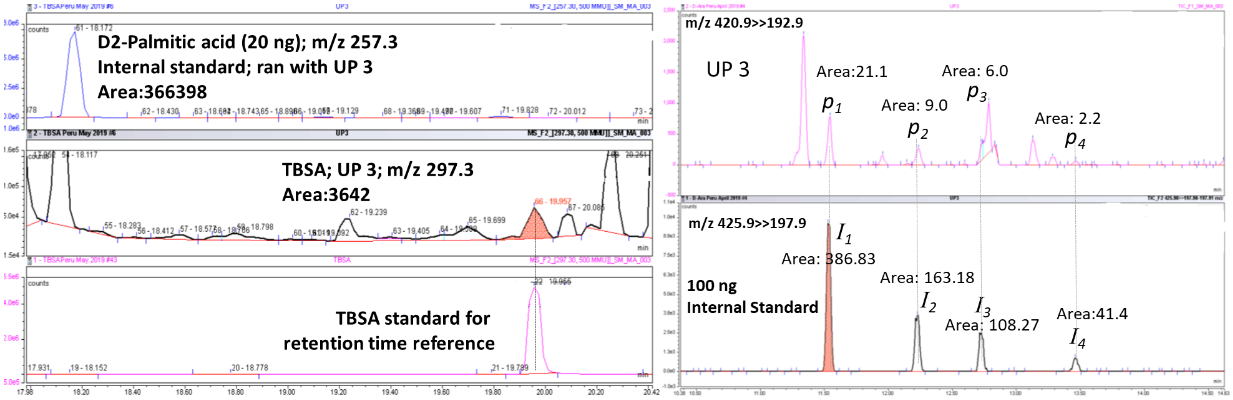

The amount of detected TBSA (T_0); I = Amount of internal standard ; 20 nano g

$$T_0 = \frac{Area\ m/z\ 297.3}{Area\ m/z\ 257.3}\ X\ I\ ng = \frac{3642}{366398}\ X\ 20\ ng = 0.198\text{ ng}$$

The mass contribution of TBSA is 298/15000 = ~0.02.

The amount of estimated LAM equivalent/mL (L_1)

For UP81, n = volume of urine; 0.7 mL

$$L_1 = \frac{T_0}{0.02\ X\ n}\frac{ng}{mL} = \frac{0.198}{0.02\ X\ 0.7}\frac{ng}{mL} = \mathbf{14.2}\text{ ng/mL}$$

Total D-Arabinose,

$$A_0 = \frac{1}{4}\left(\frac{p_1}{I_1}+\frac{p_2}{I_2}+\frac{p_3}{I_3}+\frac{p_4}{I_4}\right)X\ 10\ ng = \frac{1}{4}\left(\frac{21.1}{386.83}+\frac{9.0}{163.18}+\frac{6.0}{108.27}+\frac{2.0}{41.4}\right)X\ 100\ ng = 5.45\text{ ng}$$

The amount of LAM per milliliter (L_1, n = volume of urine used in mL; here 0.7 mL)

$$L_1 = \frac{A_0}{0.6}/(n)\text{ ng/mL} = \frac{5.45}{0.6}/(0.7) = \mathbf{13\ ng/mL}$$

Fig S13: Detection of TBSA and D-Arabinose for sample UP 3 as a representative. Respective internal standards (20 ng D2-Palmitic acid for TBSA and 100 ng 13C5-D-Arabinose for D-Arabinose assays) were used for each assay. Any loss during the process is not accounted for.
